# Isolation and Characterization of Anti-Mycobacterial Natural Products from a *Petrosia* sp. Marine Sponge

**DOI:** 10.1021/acs.jnatprod.2c01003

**Published:** 2023-03-07

**Authors:** Bhuwan Khatri Chhetri, Riya Bhanushali, Yifan Liang, Marisa R. Cepeda, Adi Kula Niradininoco, Katy Soapi, Baojie Wan, Mallique Qader, Scott G. Franzblau, Julia Kubanek

**Affiliations:** † School of Chemistry and Biochemistry, Georgia Institute of Technology, Atlanta, Georgia 30332, United States; ‡ Center for Microbial Dynamics and Infection, Georgia Institute of Technology, Atlanta, Georgia 30332, United States; § School of Biological Sciences, Georgia Institute of Technology, Atlanta, Georgia 30332, United States; ξ Institute of Applied Sciences, University of South Pacific, Suva, Fiji; γ Pacific Community, Suva, Fiji; ∇ Institute for Tuberculosis Research, College of Pharmacy, University of Illinois at Chicago, Chicago, Illinois 60612, United States; Θ Parker H. Petit Institute for Bioengineering and Bioscience, Georgia Institute of Technology, Atlanta, Georgia 30332, United States

## Abstract

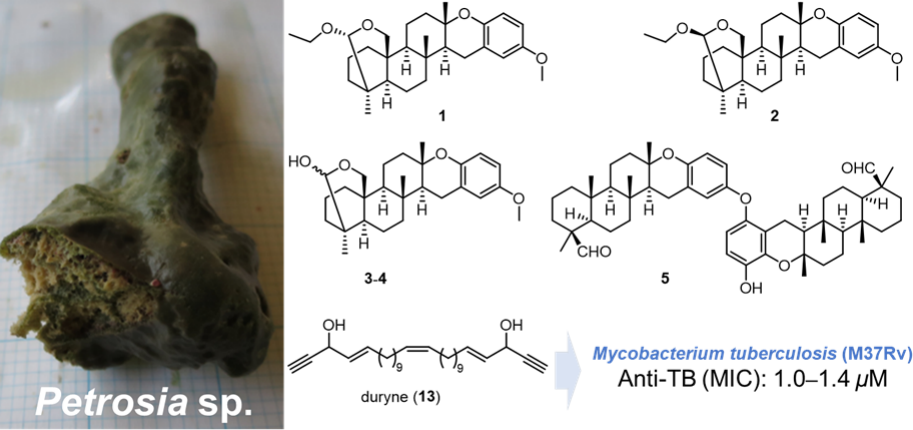

Tuberculosis (TB) is a dreadful infectious disease and a leading cause of mortality and morbidity worldwide, second in 2020 only to severe acute respiratory syndrome 2 (SARS-Cov-2). With limited therapeutic options available and a rise in multidrug-resistant tuberculosis cases, it is critical to develop antibiotic drugs that display novel mechanisms of action. Bioactivity-guided fractionation employing an Alamar blue assay for *Mycobacterium tuberculosis* strain H37Rv led to the isolation of duryne (**13**) from a marine sponge *Petrosia* sp. sampled in the Solomon Islands. Additionally, five new strongylophorine meroditerpene analogues (**1**–**5**) along with six known strongylophorines (**6**–**12**) were isolated from the bioactive fraction and characterized using MS and NMR spectroscopy, although only **13** exhibited antitubercular activity.

Tuberculosis (TB), a contagious bacterial disease caused by *Mycobacterium tuberculosis* (*Mtb*), resulted in 1.3 million mortalities in 2020 alone.^1^ More than a billion people have succumbed to TB over the past 200 years, a death toll that exceeds the combined deaths from malaria, plague, influenza, HIV/AIDS, cholera, and smallpox.^2^ While Bacillus Calmette-Guérin (BCG) is the only licensed vaccine against TB and has limited efficacy with vastly variable results (0–80% effectiveness), treatment for the active state of TB consists of an extended (6-month) regimen of first-line drugs: isoniazid, rifampicin, ethambutol, and pyrazinamide (successful in 85% of cases, data for 2019).^1^ On the other hand treatment of multidrug-resistant TB (MDR-TB) requires a second line of drugs that are comparatively less effective, have lengthy treatment regimens (up to 24 months), are more expensive, and have severe side-effects.^3^ Additionally, treatment success using second-line drugs decreases substantially for patients who are older, are further in their disease progression, are also infected with HIV, or have undergone previous TB treatment. These as well as other factors explain why MDR-TB has a low treatment success rate, posing serious risks for individual and public health.^4^

Terrestrial and marine organisms harbor a wide variety of bioactive molecules that have been the source of numerous FDA-approved drugs.^5,6^ Almost 56% of all antibacterial medications approved between 1981 and 2019 were either natural products or their derivatives. In fact, major anti-TB drugs including rifampin (belonging to the ansamycin class of polyketides) and the aminoglycosides were derived from soil bacteria *Amycolatopsis rifamycinica* and *Streptomyces griseus*, respectively.^7−9^ Several natural products have shown promising anti-TB activity and, with their mechanisms of action already elucidated, hold great potential for further development as viable drug candidates.^10−12^

Although substantial research has been devoted to development of new anti-TB agents, it is concerning that over the last three decades only two new drugs (approval of bedaquiline in 2012 by the U.S. Food and Drug Administration and delamanid in 2014 by the European Union) have been added to combat MDR-TB. Considering the promising anti-TB activity and mechanisms of action that have been revealed for several natural products, a greater interest and investment in natural products-based lead optimization may help address the health crisis caused by MDR-TB. In the current study, we explored natural products from a Solomon Islands collection of the marine sponge *Petrosia* sp.,^13−16^ leading to identification of new and known natural products with promising anti-TB profiles.

**Chart 1:**
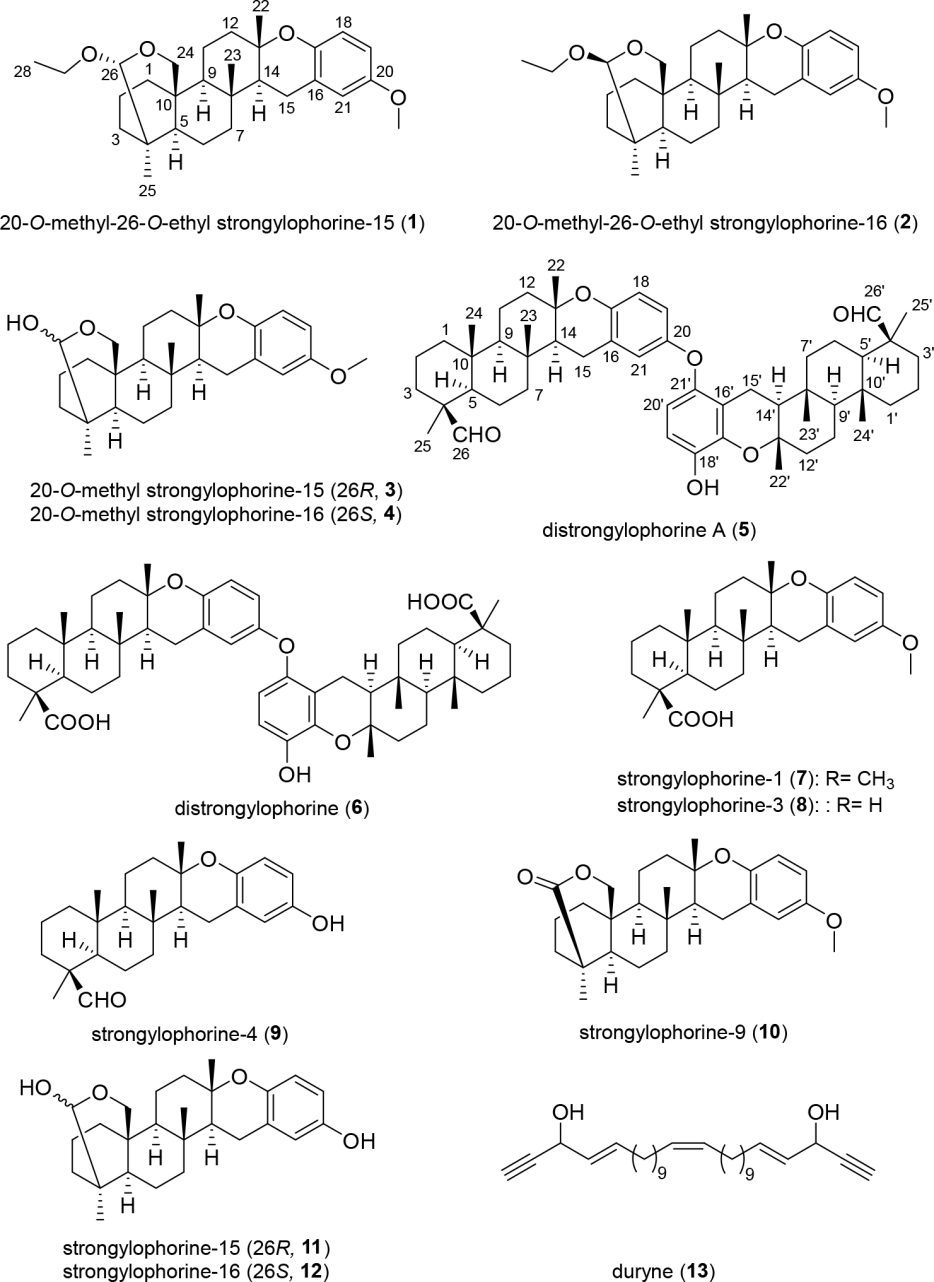


## Results and Discussion

Bioassay of several hundred marine macro-organism extracts sampled in Fiji and the Solomon Islands under the National Institutes of Health (NIH) funded International Cooperative Biodiversity Groups (ICBG) program showed that the hexanes- and CH_2_Cl_2_-soluble fractions generated from extracts of a Solomon Island marine sponge *Petrosia* sp. were bioactive against *Mtb* strain H37Rv. The bioactive hexanes- and CH_2_Cl_2_-soluble fractions were subsequently chromatographed separately on a silica gel normal phase column. Followed by an anti-TB bioassay, two chromatographic fractions were prioritized and subjected to normal phase, followed with reversed phase, HPLC separation to furnish five new strongylophorine analogues (**1**–**5**), known strongylophorines (**6**–**11**), and a known linear acetylene, **13**.

**Figure 1:**
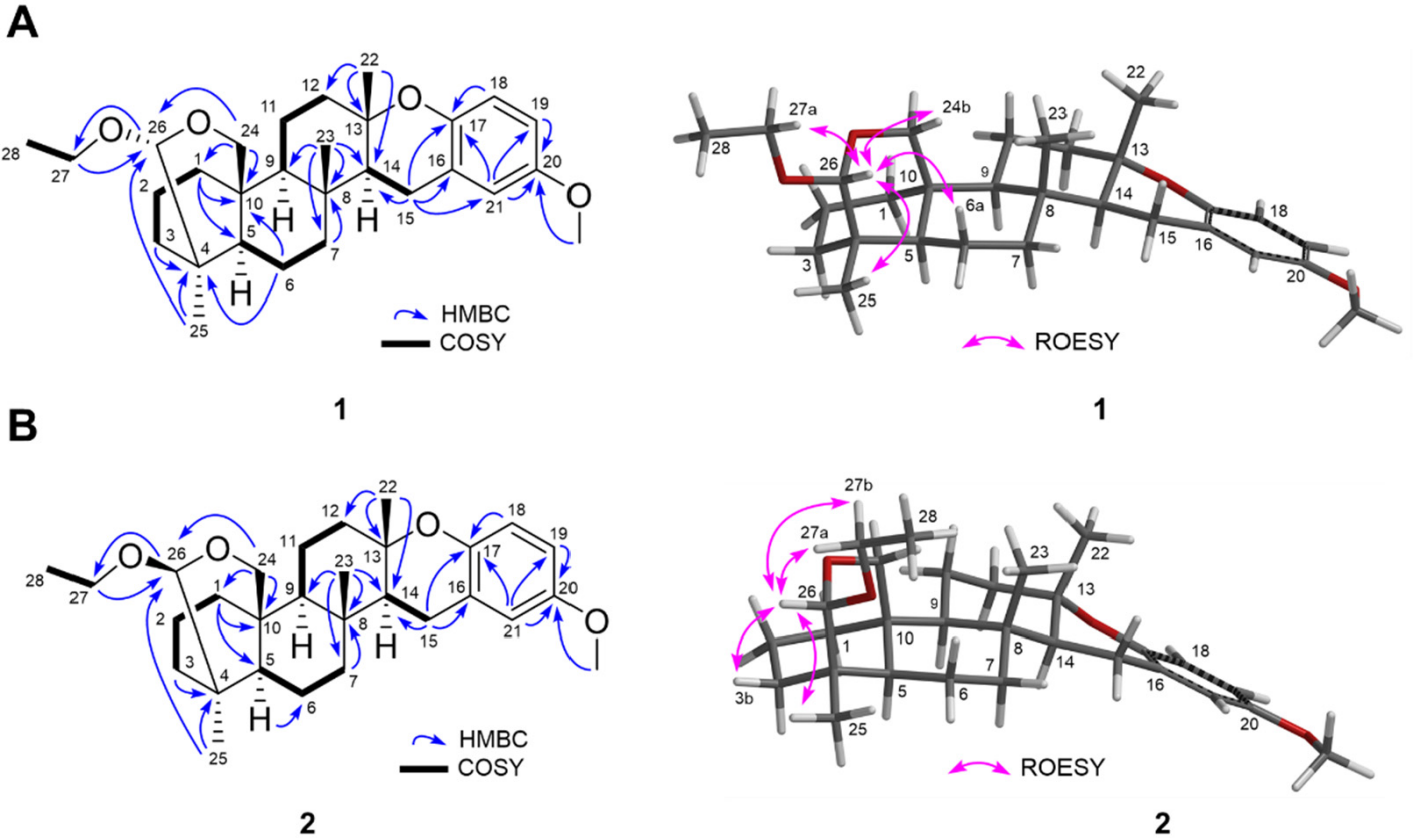
Left: Key COSY (nonwedge bold lines) and HMBC (blue arrows). Right: Key 1D ROESY (pink arrows) correlations for **1** and **2**.

20-*O*-Methyl-26-*O*-ethyl strongylophorine-15 (**1**) and 20-*O*-methyl-26-*O*-ethyl strongylophorine-16 (**2**) were isolated as colorless solids and showed ^1^H and ^13^C NMR spectroscopic features that resembled strongylophorine meroditerpene compounds previously reported from *Petrosia corticata* and *Strongylophora strongilata* (Table 1).^17,18^ High-resolution mass spectrometric (HRMS) data for **1** and **2** revealed *m*/*z* 455.3155 [M + H]^+^ and 455.3154 [M + H]^+^, respectively, suggesting a molecular formula of C_29_H_42_O_4_ for both compounds. A sharp singlet at δ_H_ 3.74 ppm (integration of 3 H atoms) observed in both ^1^H NMR spectra clearly suggested that **1** and **2** were 20-*O*-methylated analogues of 26-*O*-ethyl strongylophorine-15 and -16. The planar structures of **1** and **2** were supported by key COSY and HMBC correlations (Table 1, Figure 1). Briefly, COSY correlations were observed between H_2_-1/H_2_-2/H_2_-3, H-5/H_2_-6/H_2_-7, H-9/H_2_-11/H_2_-12, H-14/H_2_-15, and H_2_-27/H_3_-28 in both **1** and **2**. These molecular fragments could then be integrated into a pentacyclic meroditerpenoid structure (resembling the strongylophorines) with key HMBC correlations as depicted in Figure 1.^17,18^ 1D rotating frame Overhauser effect spectroscopy (1D ROESY) revealed that **1** and **2** were epimers at C-26, wherein ROESY correlations observed between H-26/H_2_-6a and 26/H_2_-24b suggested that **1** was 26*R*. Similarly, ROESY correlations observed between H-26 and H_2_-3b implied a 26*S* configuration for **2** (Figure 1).

**Table 1:** NMR Spectral Data for 20-*O*-Methyl-26-*O*-ethyl Strongylophorine-15 (**1**) and 20-*O*-Methyl-26-*O*-ethyl Strongylophorine-16 (**2**) in an 800 MHz Instrument, CDCl_3_

	**1**		**2**	
pos.	δ_C_, type	δ_H,mult_ (*J* in Hz)	δ_C_, type	δ_H,mult_ (*J* in Hz)
1a	40.5 (CH_2_)	1.12 m	40.5 (CH_2_)	1.07 m
1b		2.20 m		2.24 m
2a	22.3 (CH_2_)	1.48 m	22.4 (CH_2_)	1.52 m
2b		2.34 m		2.25 m
3a	35.4 (CH_2_)	1.08 m	40.5 (CH_2_)	1.34 m
3b		2.03 m		1.52 m
4	36.1 (C)		36.8 (C)	
5	51.5 (CH)	1.14 dd (12.6, 1.5)	48.7 (CH)	1.02 dd (13.0, 3.7)
6a	18.0 (CH_2_)	1.63 m	19.8 (CH_2_)	1.58 m
6b		1.63 m		2.17 m
7a	39.0 (CH_2_)	1.00 ddd (13.2, 13.2, 4.0)	39.1 (CH_2_)	0.91 m
7b		1.81 ddd (13.0, 3.3, 3.3)		1.75 ddd (12.8, 3.3, 3.3)
8	36.2 (C)		36.5 (C)	
9	57.0 (CH)	1.01 dd (12.5, 1.6)	57.3 (CH)	0.96 dd (12.5, 1.4)
10	37.3 (C)		37.3 (C)	
11a	19.1 (CH_2_)	1.22 m	18.9 (CH_2_)	1.24 m
11b		1.87 br d (13.8)		1.85 br d (13.8)
12a	41.8 (CH_2_)	1.61 m	41.7 (CH_2_)	1.61 m
12b		2.05 ddd (12.7, 3.2, 3.2)		2.05 ddd (12.5, 3.0, 3.0)
13	76.2 (C)		76.5 (C)	
14	52.7 (CH)	1.63 m	52.5 (CH)	1.61 m
15a	23.0 (CH_2_)	2.60 m	22.8 (CH_2_)	2.59 m
15b		2.60 m		2.59 m
16	122.6 (C)		122.9 (C)	
17	147.1 (C)		147.3 (C)	
18	117.7 (CH)	6.67 d (8.8)	117.6 (CH)	6.67 d (8.7)
19	113.2 (CH)	6.66 dd (8.9, 2.8)	113.3 (CH)	6.65 dd (8.7, 2.3)
20	153.0 (C)		153.1 (C)	
21	114.5 (CH)	6.61 d (2.6)	114.4 (CH)	6.61 d (2.3)
22	21.1 (CH_3_)	1.15 s	20.9 (CH_3_)	1.15 s
23	16.0 (CH_3_)	0.90 s	15.6 (CH_3_)	0.90 s
24a	68.0 (CH_2_)	3.58 d (12.1)	62.4 (CH_2_)	3.25 d (11.6)
24b		4.07 dd (11.6, 2.7)		4.10 dd (11.5, 2.6)
25	23.5 (CH_3_)	0.80 s	23.4 (CH_3_)	0.87 s
26	104.9 (CH)	4.43 s	104.5 (CH)	4.27 s
27a	65.2 (CH_2_)	3.47 m	62.8 (CH_2_)	3.35 m
27b		3.86 m		3.69 m
28	15.6 (CH_3_)	1.20 dd (7.0, 7.0)	15.6 (CH_3_)	1.20 dd (7.2, 7.2)
	55.9 (OCH_3_)	3.74 s	55.9 (OCH_3_)	3.74 s

**Figure 2:**
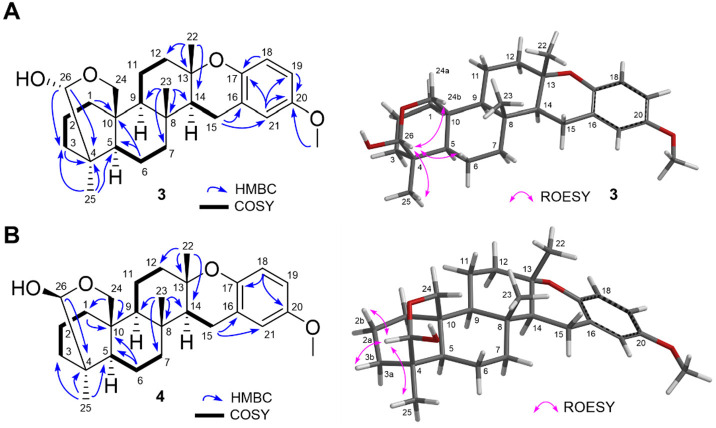
Left: Key COSY (nonwedge bold lines) and HMBC (blue arrows). Right: Key 1D ROESY (pink arrows) correlations for **3** and **4**.

20-*O*-Methyl strongylophorine-15 (**3**) and 20-*O*-methyl strongylophorine-16 (**4**) were isolated together as a mixture (Figure 2). While **3** and **4** were clearly separable in the HPLC time frame, upon drying, a 1:1 mixture was generated due to the spontaneous epimerization at C-26, as observed with ^1^H NMR spectroscopic data (Figure S15). Hence, it is likely that **1** and **2** are artifacts of the isolation process, generated from **3** and **4**, where during the rapid epimerization at C-26, the incipient oxenium ion gets captured by traces of EtOH generated from the use of EtOAc as a solvent for HPLC purification. HRMS data (*m*/*z* 427.28413 [M + H]^+^ accounting for a molecular formula of C_27_H_38_O_4_) together with ^1^H NMR spectroscopic data (sharp singlet at δ_H_ 3.74 ppm with an integration of 3 H atoms) clearly indicated that **3** and **4** were 20-*O*-methylated analogues of strongylophorine-15 (**11**) and strongylophorine-16 (**12**), respectively.^19^ The planar structure and the relative configurations at C-26 for **3** and **4** were deduced with a combination of COSY, HMBC, and 1D ROESY NMR spectroscopic data (Figure 2, Tables S1 and S2). Spin systems H_2_-1/H_2_-2/H_2_-3, H-5/H_2_-6/H_2_-7, H-9/H_2_-11/H_2_-12, and H-14/H_2_-15 observed in the COSY NMR spectroscopic data along with key HMBC correlations from the methyl protons at C-22, C-23, and C-25 supported the three cyclohexyl rings present in **3** and **4**. Down-field carbon chemical shifts (δ_C_ 76.4 and 76.5) observed for the quaternary carbons at C-13 together with a COSY correlation between H-14/H_2_-15 and HMBC correlations from H_3_-22 to C-14 and from H_2_-15 to C-16 and C-21 supported the location of the chromane moiety as observed with related strongylophorines.^17,18^ While ROESY NMR cross peaks observed between H-26/H_2_-6a and 26/H_2_-24b suggested that **3** was 26*R*, correlations observed between H-26/H_2_-3b and H-26/H_2_-3b implied a 26*S* configuration for **4** (Figure 2).

**Figure 3:**
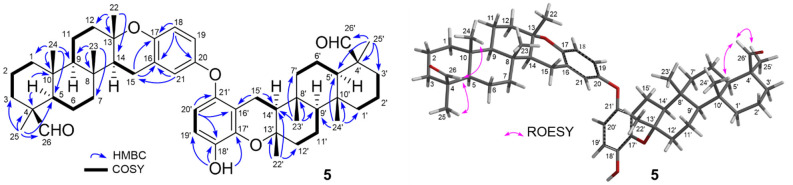
Key COSY (bold lines) and HMBC (blue arrows). Right: Key 1D ROESY (pink arrows) correlations for **5**.

Two distinctive aldehyde protons appeared as sharp singlets at 9.76 and 9.79 ppm for distrongylophorine A (**5**) and were suggestive of a new dialdehyde analogue of the strongylophorine dimer, distrongylophorine (**6**).^14^ Further interpretation with HMBC NMR spectroscopic data clearly suggested that the two aldehyde functionalities were positioned at C-26 and C-26′ (Figure 3). The relative configurations of the aldehyde functionalities were revealed with 1D ROESY experiments where a ROESY correlation was observed between H-24/H-26 and H-24′/ H-26′. ^1^H and ^13^C NMR chemical shifts for **5**, along with their corresponding COSY and HMBC correlations, have been reported in Table S3. Finally, the identities of known natural products **6**–**13** were confirmed by comparing their HRMS and ^1^H and ^13^C data with existing literature.^13−15,19^

Assessment of pure **1**, **3**, **4**, **7**–**10**, and **13** in an Alamar blue assay (MABA) showed that **13** was potently active against the virulent strain (H37Rv) of *Mtb* with an MIC of 1.4 μM, comparable to the tuberculosis drug linezolid (1.0 μM, Table 2).^20^ The strongylophorines did not show significant inhibition at a test concentration of 25 μg/mL. As MABA represents the rapidly growing state of *Mtb* and does not recapitulate the nonreplicating persistence physiological state of the bacteria, a low-oxygen recovery assay (LORA) was also employed.^21−23^ The nonreplicating persistence physiological state of the bacteria is a critical issue for all tuberculosis medications, requiring treatments that last several months, and hence represents a crucial phenotype to be targeted in the development of new drugs. Interestingly, duryne (**13**) retained its promising bioactivity in a LORA assay as well and had a MIC of 1.0 μM (comparable with the MIC for linezolid and pretomanid, Table 2). However, **13** was not active against *M. abscessus* and *M. avium* (data not shown). Its IC_50_ of 0.4 μM against cultured Vero cells was consistent with previous reports of cytotoxicity in P388 murine leukemia, colon (HCT-8), lung (A549), mammary (MCF7), and HeLa cervical cells (IC_50_ ∼0.2 μM).^15,16^ The similar potency of **13** in MABA and LORA assays (Table 2), combined with toxicity toward Vero cells, is consistent with the possibility that **13** could be targeting the electron transport chain or a conserved cell membrane component, rather than acting on cell wall synthesis. Activity of **13** against *M. tuberculosis* but not *M. abscessus* or *M. avium* is not predictive of mechanism of action because such MIC differences can be due to any number of factors including cell differences in penetration, efflux, metabolism, or activation of the target itself. It is worth noting that structure–activity relationship studies on molecules similar to duryne have shown that the lipid chain length, stereochemistry of the propargyl alcohol, and the presence of one or multiple acetylenic groups affect their cytotoxicity.^24,25^ Structural analogues of **13** with additional hydroxyl or carboxylic acid moieties (e.g., pellynols, neopetroformynes, petroformynic acids) have been shown to retain their cytotoxicity, generating hope that issues can be addressed related to druggability and solubility of these hydrophobic molecules.^26^ In conclusion, considering the low success rate on finding new molecules that inhibit *Mtb*, **13** and its analogues could be explored further to determine whether the antitubercular and cytotoxic effects of **13** can be decoupled through medicinal chemistry efforts.^27,28^

**Table 2:** Anti-TB Activity of Duryne (**13**) and Some Clinically Used Anti-TB Drugs

	MIC (μM)^a^	
compound	MABA assay^b^	LORA assay^c^
duryne (**13**)	1.4 ± 0.0	1.0 ± 0.1
linezolid	1.0	1.7
pretomanid	0.2	2.0
isoniazid	0.2	>128
rifampin	0.04	0.09
bedaquiline	0.03	0.1

a Average of two independent experiments with standard error of the mean for **13**.

b MABA: Alamar blue assay.

c LORA: low-oxygen recovery assay.

## Experimental Section

### General Experimental Procedures

All NMR spectroscopic data (1D and 2D) were acquired on an 18.8 T (800 MHz for ^1^H and 201 MHz for ^13^C) Bruker Avance IIIHD instrument equipped with a 3 mm triple resonance cryoprobe or a 16.4 T (700 MHz for ^1^H and 175 MHz for ^13^C) Bruker Avance IIIHD instrument with a 5 mm indirect broadband cryoprobe. CDCl_3_ or pyridine-*d*_5_ was used as the NMR solvent for all samples and referenced to residual solvent peaks δ_H_ 7.26, δ_C_ 77.2 for CDCl_3_ and δ_H_ 7.22, δ_C_ 123.9 for pyridine-*d*_5_. NMR spectroscopic data were processed and analyzed using MestReNova 11.0.4.

High-resolution mass spectrometric data were acquired in a Thermo Scientific Orbitrap ID-X instrument. Both normal and reversed phase HPLC chromatographic separations were performed using a Waters 1525 binary pump coupled with a Waters 2996 photodiode array detector and an Altech 800 evaporative light scattering detector.

### Specimen Collection and Species Identification

The marine sponge *Petrosia* sp. (G-1323) was collected in 2016 off Kuri Point near Munda, New Georgia Island, Solomon Islands (S 8°20′15.7″, E 157°13′44.4″) at a depth of 17 m. It was found growing on a reef slope, appeared as fingerlike projections, and had no centrangulates. The sponge sample had a hard texture with a greenish gray exterior and a beige-colored inside (Figure S1). Morphological and DNA voucher samples were deposited in formalin and molecular grade EtOH, respectively, and stored at University of South Pacific, Suva, Fiji. The sponge genus was assigned by morphological and chemotaxonomic comparison with the literature.^29,30^

### Isolation of Natural Products

*Petrosia* sp. (1000 g wet weight) was extracted with CH_2_Cl_2_ and MeOH to furnish 11.8 g of crude extract. Liquid/liquid partitioning of the extract between 1:9 H_2_O/MeOH and hexanes furnished 0.5 g (F1) of hexanes-soluble fraction. The aqueous layer was adjusted to 2:3 H_2_O/MeOH and partitioned with CH_2_Cl_2_, resulting in 8.0 g (F2) of CH_2_Cl_2_-soluble fraction. Finally, the MeOH/H_2_O layer was partially evaporated to remove all traces of MeOH, and the remaining aqueous solution was partitioned with *n-*butanol to furnish 0.4 g (F3) of *n-*butanol-soluble fraction. The hexanes-soluble fraction (F1) was subjected to normal phase silica gel column chromatography (17 g of 230–400 mesh silica gel) eluting with hexanes to EtOAc (step gradient), followed with EtOAc to MeOH (step gradient) to furnish 17 (F1.1–F1.17) fractions. Similarly, 2.1 g of CH_2_Cl_2_-soluble fraction (F2) was also subjected to normal phase silica gel column chromatography (55 g of 230–400 mesh silica gel) with hexanes to EtOAc (step gradient), followed with EtOAc to MeOH (step gradient) to furnish 11 (F2.1–F2.11) fractions. Fractions F1.5 (78 mg) and F2.4 (365 mg) resulting from the above-mentioned silica gel column-based fractionation of the hexanes- and CH_2_Cl_2_-soluble liquid/liquid partition fractions, respectively, were prioritized for further separation based on their promising anti-TB activity on a MABA *Mtb* bioassay.

F1.5 (40 mg) was separated in a normal phase Luna (5 μm, 100 Å, 4.6 × 250 mm) silica HPLC column using isocratic 1:9 EtOAc/hexanes over 5 min, followed with a gradient of 1:9 EtOAc/hexanes to 1:1 EtOAc/CH_2_Cl_2_ over 20 min (solvent flow rate = 1 mL/min). Eighteen fractions were generated (F1.5.1–F1.5.18) of which F1.5.3–F1.5.7 (*t*_R_ = 5–9.5 min) contained pure **1**, while F1.5.8–F1.5.9 (*t*_R_ = 9.5–11.5 min) had partially pure **1**. F1.5.3 was quantified with qNMR (0.22 mg of **1**, 0.00004% w/wet weight isolated yield) and used for bioassay. A portion (∼0.4 mg) of F1.5.5 (∼0.5 mg) was subjected to reversed phase HPLC separation (ZORBAX SB-C18, 5 μm, 4.6 × 250 mm column) starting with a gradient of 1:9 H_2_O/MeCN to 1:1 ratio of 1:9 H_2_O/MeCN and 1:9 MeCN/iPrOH over 12 min, followed with isocratic 1:9 MeCN/iPrOH for 6 min (solvent flow rate = 1.0 mL/min) and eluted **1** and **2** at 14.6 and 12.6 min, respectively. While NMR and HRMS characterization data for **2** were acquired immediately after isolation, quantification of **2** for bioassay was not feasible due to compound degradation issues when stored over a week. A portion (∼1 mg) of F1.5.16 (eluted between 16 and 17 min in the preceding HPLC separation, ∼2 mg) was subjected to reversed phase HPLC separation (ZORBAX SB-C18, 5 μm, 4.6 × 250 mm column, monitored with ELSD) with a gradient of 1:9 H_2_O/MeCN to 1:1 ratio of 1:9 H_2_O/MeCN and 1:9 MeCN/iPrOH over 15 min eluting **13** (0.13 mg, 0.00004% w/wet weight isolated yield, quantified with qNMR and used for bioassay) at 12.3 min (solvent flow rate = 1.0 mL/min).

F1.5 (38 mg) was subjected to semipreparative (Alltima Silica, 5 μm, 250 × 10 mm) silica gel HPLC column-based separation using isocratic 1:9 EtOAc/hexanes over 3 min, followed with a gradient of 1:9 EtOAc/hexanes to 1:1 EtOAc/CH_2_Cl_2_ over 7 min and isocratic 1:1 EtOAc/CH_2_Cl_2_ for 20 min (solvent flow rate = 2.0 mL/min) to generate 34 fractions (F1.5.1′–F1.5.34′). A portion of fraction F1.5.19′ (eluted between 16.0 and 16.5 min in the preceding semipreparative HPLC separation step) was purified on a ZORBAX SB-C18, 5 μm, 4.6 × 250 mm HPLC column with solvent gradient starting at 1:9 H_2_O/MeCN to a 1:1 ratio of 1:9 H_2_O/MeCN and 1:9 MeCN/iPrOH over 15 min (solvent flow rate = 1 mL/min) to isolate an epimeric mixture of **3** and **4** (0.18 mg, 0.00008% w/wet weight isolated yield, quantified with qNMR and used for bioassay) at 8.5 min. A portion (∼0.9 mg) of F1.5.15′ (∼2.2 mg) was subjected to HPLC purification on a ZORBAX SB-C18, 5 μm, 4.6 × 250 mm column using a solvent gradient of 1:9 H_2_O/MeCN to 1:1 ratio of 1:9 H_2_O/MeCN and 1:9 MeCN/iPrOH over 15 min (solvent flow rate = 1 mL/min), leading to the elution of **7** (0.32 mg, 0.0002% w/wet weight isolated yield, quantified with qNMR and used for bioassay) at 7.6 min. A portion of F1.5.18′ (eluted between 15.4 and 16.0 min in the preceding semipreparative HPLC separation step and weighed ∼5.9 mg on an analytical balance, 0.001% w/wet weight isolated yield) was quantified with qNMR (contained 1.7 mg of **10**) and used for bioassay.

Fraction F2.4 (163 mg) was subjected to normal phase semipreparative HPLC separation on a Alltima silica, 5 μm, 10 × 250 mm column using isocratic 1:9 EtOAc/hexanes for 5 min (solvent flow rate = 2 mL/min), followed with a gradient of 1:9 EtOAc/hexanes to 100% 1:1 EtOAc/CH_2_Cl_2_ over 15 min. Isocratic 1:1 EtOAc/CH_2_Cl_2_ was maintained for 10 min, followed with a gradient of 1:1 EtOAc/CH_2_Cl_2_ to 100% 1:9 EtOAc/hexanes over 2 min. Isocratic flow of 1:9 EtOAc/hexanes was retained for 8 min and generated 36 fractions (F2.4.1–F2.4.36). While the epimeric mixture of **11** and **12** eluted in fractions F2.4.25– F2.4.28 (eluting between 24.5 and 26 min), further purification and quantification of **11** and **12** were not feasible due to degradation issues. A portion (∼0.9 mg) of F2.4.16 (∼3.6 mg) was subjected to reversed phase HPLC (ZORBAX SB-C18, 5 μm, 4.6 × 250 mm column) using a solvent gradient of 1:9 H_2_O/MeCN to a 1:1 ratio of 1:9 H_2_O/MeCN and 1:9 MeCN/iPrOH over 12 min, followed with an isocratic 1:1 ratio of 1:9 H_2_O/MeCN and 1:9 MeCN/iPrOH for 6 min eluting **5** at 14.4 min (**5** was not subjected to quantification or bioassay due to degradation issues). A part (∼2.9 mg) of F2.4.19 (eluted between 16.0 and 16.5 min in the preceding semipreparative HPLC separation step, 47 mg) was subjected to a ZORBAX SB-C18, 5 μm, 4.6 × 250 mm HPLC column separation using a solvent gradient of 1:9 H_2_O/MeCN to 1:1 ratio of 1:9 H_2_O/MeCN and 1:9 MeCN/iPrOH over 12 min (solvent flow rate = 1 mL/min), giving **8** (*t*_R_ = 4.3 min, 0.54 mg, 0.008% w/wet weight isolated yield, quantified with qNMR and used for bioassay) and **6** (*t*_R_ = 10.8 min, 0.44 mg, 0.008% w/wet weight isolated yield, quantified with qNMR), respectively. Distrongylophorine (**6**) was not subjected to bioassay due to degradation issues. A portion (∼1.3 mg) of F2.4.16 (∼3.6 mg) was subjected to reversed phase HPLC (ZORBAX SB-C18, 5 μm, 4.6 × 250 mm column) using a solvent gradient of 1:9 H_2_O/MeCN to 1:1 ratio of 1:9 H_2_O/MeCN and 1:9 MeCN/iPrOH over 12 min (solvent flow rate = 1 mL/min), eluting **9** (0.14 mg, 0.0003% w/wet weight isolated yield, quantified with qNMR and used for bioassay) at 6.0 min.

#### 20-*O*-Methyl-26-*O*-ethyl strongylophorine-15 (**1**)

Colorless solid; ^1^H and ^13^C NMR data, Table 1; HRMS *m*/*z* [M + H]^+^ calcd for C_29_H_43_O_4_^+^ 455.3156, found 455.3155.

#### 20-*O*-Methyl-26-*O*-ethyl strongylophorine-16 (**2**)

Colorless solid; ^1^H and ^13^C NMR data, Table 1; HRMS *m*/*z* [M + H]^+^ calcd for C_29_H_43_O_4_^+^ 455.3156, found 455.3154.

#### 20-*O*-Methyl strongylophorine-15 and -16 (**3** and **4**)

White amorphous solid; ^1^H and ^13^C NMR data, Tables S1 and S2; HRMS *m*/*z* [M + H]^+^ calcd for C_27_H_39_O_4_^+^ 427.2843, found 427.2841.

#### Distrongylophorine A (**5**)

White amorphous solid; ^1^H and ^13^C NMR data, Table S3; HRMS *m*/*z* [M + H]^+^ calcd for C_52_H_71_O_6_^+^ 791.5246, found 791.5245.

#### Duryne (**13**)

Yellow oil; ^1^H NMR (800 MHz, CDCl_3_) δ_H_ 5.91 (ddd, *J* = 15.2, 6.7, 1.0 Hz, 2H), 5.61 (dddd, *J* = 15.1, 6.2, 1.4, 1.4 Hz, 2H), 5.35 (m, 2H), 4.84 (m, 2H), 2.56 (d, *J* = 2.2 Hz, 2H), 2.07 (m, 4H), 2.01 (m, 4H), 1.39 (m, 4H), 1.33 (m, 4H), 1.27 (br s, 20H); ^13^C NMR (201 MHz, CDCl_3_) 134.8, 134.8, 130.0, 130.0, 128.5, 128.5, 83.5, 83.5, 74.1, 74.1, 63.0, 63.0, 32.1, 32.1, 29.9, 29.9, 29.7, 29.7, 29.7, 29.7, 29.6, 29.6, 29.5, 29.5, 29.3, 29.3, 29.0, 29.0, 27.4, 27.4; HRMS *m*/*z* [M + H]^+^ calcd for C_30_H_49_O_2_^+^ 441.3728, found 441.3725.^15^

### Antimycobacterial Bioassay

The 96-well microplate Alamar blue assay and low-oxygen recovery assay to assess anti-TB activity were conducted using the established protocol. While MABA is a quantitative assay that evaluates drug susceptibility of any replicating *Mtb* strain, the LORA assay mimics hypoxic conditions by using a recombinant strain of auto-bioluminescent *M. tuberculosis* strain that expresses the entire Lux operon (luxCDABE).^23,31^ A virulent strain of *Mtb* (H37Rv) was used for the MABA assay. MIC was defined as the lowest concentration of an extract or pure compound needed to observe *a* ≥ 90% inhibition of fluorescence (MABA) or luminescence (LORA). Inhibition of *M. abscessus* (ATCC19977) was tested following the MABA assay procedure and incubated for 3 days at 37 °C. *M. avium* (ATCC15769) was cultured in 7H9 medium supplemented with 10% OADC and incubated for 6 days at 37 °C. At the end of the incubation 0.6 mM resazurin with Tween 80 was added and the fluorescence was measured after 4 (*M. abscessus*) and 24 (*M. avium*) hours at excitation/emission wavelengths of 530/590 nm to calculate the MIC.^32^ While linezolid, pretomanid, isoniazid, rifampin, and bedaquiline were positive controls, DMSO (vehicle solvent) was used as a negative control.

### Cytotoxicity Assay

Green monkey kidney cells (Vero cell CCL-81) were used to assess the cytotoxicity. Cultured Vero cells in Eagle’s minimum essential medium (MEM) containing 10% fetal bovine serum (FBS) supplemented with penicillin and streptomycin antibiotic mix were used. The cells were microscopically quantified into a density of 2 × 10^5^ cells/mL. A 100 μL portion of cell suspension was added to the test compounds and incubated for 3 days at 5% CO_2_ and 37 °C. At the end of the incubation period 20 μL of 0.6 mM resazurin was added, and the fluorescence readout was measured after 4 h of incubation to calculate IC_50_.^33^

## Supporting Information Available

Additional specimen collection and natural product characterization data (PDF)

## Dedication

Dedicated to Dr. Mary J. Garson, The University of Queensland, for her pioneering work on bioactive natural products.

## References

[ref1] WHO Global tuberculosis report 2021. https://www.who.int/publications/i/item/9789240037021 (accessed 2022–06–15).

[ref2] Paulson T. Epidemiology: A mortal foe. Nature. 2013, 502 (7470), S2–S3. 10.1038/502S2a. 24108078

[ref3] Harries A. D.; Lin Y.; Kumar A. M.; Satyanarayana S.; Takarinda K. C.; Dlodlo R. A.; Zachariah R.; Olliaro P. What can National TB Control Programmes in low-and middle-income countries do to end tuberculosis by 2030? F1000Research 2018, 7, 1011 10.12688/f1000research.14821.1. PMC6039935 30026917

[ref4] Falzon D.; Gandhi N.; Migliori G. B.; Sotgiu G.; Cox H. S.; Holtz T. H.; Hollm-Delgado M. G.; Keshavjee S.; DeRiemer K.; Centis R.; D’Ambrosio L.; Lange C. G.; Bauer M.; Menzies D. Resistance to fluoroquinolones and second-line injectable drugs: impact on multidrug-resistant TB outcomes. Eur. Respir. J. 2013, 42 (1), 156–168. 10.1183/09031936.00134712. 23100499 PMC4487776

[ref5] Newman D. J.; Cragg G. M. Natural Products as Sources of New Drugs over the Nearly Four Decades from 01/1981 to 09/2019. J. Nat. Prod. 2020, 83 (3), 770–803. 10.1021/acs.jnatprod.9b01285. 32162523

[ref6] Lu W. Y.; Li H. J.; Li Q. Y.; Wu Y. C. Application of marine natural products in drug research. Bioorg. Med. Chem. 2021, 35, 116058 10.1016/j.bmc.2021.116058. 33588288

[ref7] Sensi P. History of the development of rifampin. Rev. Infect. Dis. 1983, 5, S402–S406. 10.1093/clinids/5.Supplement_3.S402. 6635432

[ref8] Bala S.; Khanna R.; Dadhwal M.; Prabagaran S. R.; Shivaji S.; Cullum J.; Lal R. Reclassification of *Amycolatopsis mediterranei* DSM 46095 as *Amycolatopsis rifamycinica* sp. nov. Int. J. Syst. Evol. 2004, 54 (4), 1145–1149. 10.1099/ijs.0.02901-0. 15280283

[ref9] Wainwright M. Streptomycin: discovery and resultant controversy. Hist. Philos. Life Sci. 1991, 13 (1), 97–124. 1882032

[ref10] Han J.; Liu X.; Zhang L.; Quinn R. J.; Feng Y. Anti-mycobacterial natural products and mechanisms of action. Nat. Prod. Rep. 2022, 39 (1), 77–89. 10.1039/D1NP00011J. 34226909

[ref11] Cazzaniga G.; Mori M.; Chiarelli L. R.; Gelain A.; Meneghetti F.; Villa S. Natural products against key *Mycobacterium tuberculosis* enzymatic targets: Emerging opportunities for drug discovery. Eur. J. Med. Chem. 2021, 224, 113732 10.1016/j.ejmech.2021.113732. 34399099

[ref12] Hou X.-M.; Wang C.-Y.; Gerwick W. H.; Shao C.-L. Marine natural products as potential anti-tubercular agents. Eur. J. Med. Chem. 2019, 165, 273–292. 10.1016/j.ejmech.2019.01.026. 30685527

[ref13] Yu W.; Hjerrild P.; Overgaard J.; Poulsen T. B. A Concise Route to the Strongylophorines. Angew. Chem. Int. Ed. 2016, 55 (29), 8294–8438. 10.1002/anie.201602476. 27239800

[ref14] Balbin-Oliveros M.; Edrada R. A.; Proksch P.; Wray V.; Witte L.; Van Soest R. W. A new meroditerpenoid dimer from an undescribed Philippine marine sponge of the genus. Strongylophora. J. Nat. Prod. 1998, 61 (7), 948–952. 10.1021/np980005y. 9677282

[ref15] Hitora Y.; Takada K.; Okada S.; Ise Y.; Matsunaga S. (−)-Duryne and its homologues, cytotoxic acetylenes from a marine Sponge *Petrosia* sp. J. Nat. Prod. 2011, 74 (5), 1262–1267. 10.1021/np200271n. 21534590

[ref16] Wright A. E.; McConnell O. J.; Kohmoto S.; Lui M. S.; Thompson W.; Snader K. M. Duryne, a new cytotoxic agent from the marine sponge *Cribrochalina dura*. Tetrahedron Lett. 1987, 28 (13), 1377–1380. 10.1016/S0040-4039(00)95931-8.

[ref17] Lee J. S.; Abdjul D. B.; Yamazaki H.; Takahashi O.; Kirikoshi R.; Ukai K.; Namikoshi M. Strongylophorines, new protein tyrosine phosphatase 1B inhibitors, from the marine sponge *Strongylophora strongilata* collected at Iriomote Island. Bioorg. Med. Chem. Lett. 2015, 25 (18), 3900–3902. 10.1016/j.bmcl.2015.07.039. 26253631

[ref18] Noda A.; Sakai E.; Kato H.; Losung F.; Mangindaan R. E.; de Voogd N. J.; Yokosawa H.; Tsukamoto S. Strongylophorines, meroditerpenoids from the marine sponge *Petrosia corticata*, function as proteasome inhibitors. Bioorg. Med. Chem. Lett. 2015, 25 (13), 2650–2653. 10.1016/j.bmcl.2015.04.075. 25987373

[ref19] Liu H.; Namikoshi M.; Akano K.; Kobayashi H.; Nagai H.; Yao X. Seven new meroditerpenoids, from the marine sponge *Strongylophora strongylata*, that inhibited the maturation of starfish oocytes. J. Asian Nat. Prod. Res. 2005, 7 (4), 661–670. 10.1080/1028602032000169604. 16087642

[ref20] Collins L.; Franzblau S. G. Microplate alamar blue assay versus BACTEC 460 system for high-throughput screening of compounds against *Mycobacterium tuberculosis* and *Mycobacterium avium*. Antimicrob. Agents Chemother. 1997, 41 (5), 1004–1009. 10.1128/AAC.41.5.1004. 9145860 PMC163841

[ref21] Cho S. H.; Warit S.; Wan B.; Hwang C. H.; Pauli G. F.; Franzblau S. G. Low-oxygen-recovery assay for high-throughput screening of compounds against nonreplicating *Mycobacterium tuberculosis*. Antimicrob. Agents Chemother. 2007, 51 (4), 1380–1385. 10.1128/AAC.00055-06. 17210775 PMC1855511

[ref22] Hwang J. M.; Oh T.; Kaneko T.; Upton A. M.; Franzblau S. G.; Ma Z.; Cho S. N.; Kim P. Design, synthesis, and structure-activity relationship studies of tryptanthrins as antitubercular agents. J. Nat. Prod. 2013, 76 (3), 354–367. 10.1021/np3007167. 23360475

[ref23] Cho S.; Lee H. S.; Franzblau S. Microplate Alamar Blue Assay (MABA) and Low Oxygen Recovery Assay (LORA) for *Mycobacterium tuberculosis*. Methods Mol. Biol. 2015, 1285, 281–292. 10.1007/978-1-4939-2450-9_17. 25779323

[ref24] El Arfaoui D.; Listunov D.; Fabing I.; Oukessou M.; Frongia C.; Lobjois V.; Samson A.; Ausseil F.; Ben-Tama A.; El Hadrami E. M. Identification of Chiral Alkenyl-and Alkynylcarbinols as Pharmacophores for Potent Cytotoxicity. ChemMedChem. 2013, 8 (11), 1779–1786. 10.1002/cmdc.201300230. 24014463

[ref25] Bourkhis M.; Gaspard H.; Rullière P.; De Almeida D. K.; Listunov D.; Joly E.; Abderrahim R.; de Mattos M. C.; De Oliveira M. C.; Maraval V. Skeletal Optimization of Cytotoxic Lipidic Dialkynylcarbinols. ChemMedChem. 2018, 13 (11), 1124–1130. 10.1002/cmdc.201800118. 29603643

[ref26] Lee Y. J.; Cho Y.; Tran H. N. K. Secondary Metabolites from the Marine Sponges of the Genus *Petrosia*: A Literature Review of 43 Years of Research. Mar. Drugs. 2021, 19 (3), 122 10.3390/md19030122. 33668842 PMC7996255

[ref27] Simithy J.; Fuanta N. R.; Alturki M.; Hobrath J. V.; Wahba A. E.; Pina I.; Rath J.; Hamann M. T.; DeRuiter J.; Goodwin D. C.; Calderón A. I. Slow-Binding Inhibition of *Mycobacterium tuberculosis* Shikimate Kinase by Manzamine Alkaloids. Biochemistry 2018, 57 (32), 4923–4933. 10.1021/acs.biochem.8b00231. 30063132 PMC12570217

[ref28] Zhang F.; Zhao M.; Braun D. R.; Ericksen S. S.; Piotrowski J. S.; Nelson J.; Peng J.; Ananiev G. E.; Chanana S.; Barns K.; Fossen J.; Sanchez H.; Chevrette M. G.; Guzei I. A.; Zhao C.; Guo L.; Tang W.; Currie C. R.; Rajski S. R.; Audhya A.; Andes D. R.; Bugni T. S. A marine microbiome antifungal targets urgent-threat drug-resistant fungi. Science. 2020, 370 (6519), 974–978. 10.1126/science.abd6919. 33214279 PMC7756952

[ref29] Lee Y. J.; Cho Y.; Tran H. N. K. Secondary Metabolites from the Marine Sponges of the Genus *Petrosia*: A Literature Review of 43 Years of Research. Mar. Drugs. 2021, 19 ( (3), ), 122 10.3390/md19030122. 33668842 PMC7996255

[ref30] Zea S.; Henkel T. P.; Pawlik J. R. 2014. The Sponge Guide: A Picture Guide to Caribbean Sponges, 3rd ed.; Available online at www.spongeguide.org. (accessed 2022–06–26).

[ref31] Shetye G. S.; Choi K. B.; Kim C. Y.; Franzblau S. G.; Cho S. *In Vitro* Profiling of Antitubercular Compounds by Rapid, Efficient, and Nondestructive Assays Using Autoluminescent *Mycobacterium tuberculosis*. Antimicrob. Agents Chemother. 2021, 65 (8), e0028221 10.1128/AAC.00282-21. 34097493 PMC8284454

[ref32] Gao W.; Kim J.-Y.; Anderson J. R.; Akopian T.; Hong S.; Jin Y.-Y.; Kandror O.; Kim J.-W.; Lee I.-A.; Lee S.-Y.; et al. The cyclic peptide ecumicin targeting ClpC1 is active against *Mycobacterium tuberculosis In Vivo*. Antimicrob. Agents Chemother. 2015, 59 (2), 880–889. 10.1128/AAC.04054-14. 25421483 PMC4335914

[ref33] Nandikolla A.; Srinivasarao S.; Khetmalis Y. M.; Kumar B. K.; Murugesan S.; Shetye G.; Ma R.; Franzblau S. G.; Sekhar K. V. G. C. Design, synthesis and biological evaluation of novel 1,2,3-triazole analogues of Imidazo-[1,2-a]-pyridine-3-carboxamide against *Mycobacterium tuberculosis*. Toxicol. In Vitro. 2021, 74, 105137 10.1016/j.tiv.2021.105137. 33684466

