# Supplementary material for: Isolation and Characterization of Anti-Mycobacterial Natural Products from a Petrosia sp. Marine Sponge
Source: J Nat Prod. 2023 Mar 7;86(3):574–81. doi: 10.1021/acs.jnatprod.2c01003 (PMC10043868; doi:10.1021/acs.jnatprod.2c01003)
Supplement: Supplementary file 1 — np2c01003_si_001.pdf [file np2c01003_si_001.pdf]

Supplementary Information

Isolation and Characterization of Anti-Mycobacterial Natural Products from a *Petrosia* sp. Marine Sponge

Bhuwan Khatri Chhetri,[†] Riya Bhanushali,[§] Yifan Liang,[†] Marisa Cepeda,[†] Adi Kula Niradininoco[ξ], Katy Soapi,[ξ, ɣ] Baojie Wan,[∇] Mallique Qader, [∇] Scott G. Franzblau,[∇] and Julia Kubanek[*,†,‡,§,Θ]

[†] School of Chemistry and Biochemistry, Georgia Institute of Technology, Atlanta, GA 30332, United States

[‡] Center for Microbial Dynamics and Infection, Georgia Institute of Technology, Atlanta, GA 30332, United States

[§] School of Biological Sciences, Georgia Institute of Technology, Atlanta, GA 30332, United States

[ξ] Institute of Applied Sciences, University of South Pacific, Suva, Fiji

[ɣ] Pacific Community, Suva, Fiji

[∇] Institute for Tuberculosis Research, College of Pharmacy, University of Illinois at Chicago, Chicago, IL 60612, United States

[Θ] Parker H. Petit Institute for Bioengineering and Bioscience, Georgia Institute of Technology, Atlanta, GA 30332, United States

[*] Author to whom correspondence should be addressed.

Contents

Figure S1. Collection photo for *Petrosia* sp. marine sponge 3
Characterization data for distrongylophorine (**6**) 3
Characterization data for strongylophorine-1 (**7**) 3
Characterization data for strongylophorine-3 (**8**) 3
Characterization data for strongylophorine-4 (**9**) 4
Characterization data for strongylophorine-9 (**10**) 4
Characterization data for strongylophorine-15 (**11**) and strongylophorine-16 (**12**) 4
Table S1. NMR spectral data for 20-*O*-methyl strongylophorine-15 (**3**) in 800 MHz instrument, $CDCl_3$, isolated as a mixture with 20-*O*-methyl strongylophorine-16 (**4**). 5
Table S2. NMR spectral data for 20-*O*-methyl strongylophorine-16 (**4**) in 800 MHz instrument, $CDCl_3$, isolated as a mixture with 20-*O*-methyl strongylophorine-15 (**3**). 6
Table S3: NMR spectral data for distrongylophorine A (**5**) in 800 MHz instrument, $CDCl_3$ 7
Figure S2. ^1H NMR spectrum of **1** in 800 MHz instrument, $CDCl_3$ 8
Figure S3. HSQC NMR spectrum of **1** in 800 MHz instrument, $CDCl_3$ 8
Figure S4. COSY NMR spectrum of **1** in 800 MHz instrument, $CDCl_3$ 9
Figure S5. HMBC NMR spectrum of **1** in 800 MHz instrument, $CDCl_3$ 9
Figure S6. 1D ROESY NMR spectrum of **1** in 800 MHz instrument, $CDCl_3$ 10
Figure S8. ^1H NMR spectrum of **2** in 800 MHz instrument, $CDCl_3$ 11
Figure S9. ^{13}C NMR spectrum of **2** in 800 MHz instrument, $CDCl_3$ 11
Figure S10. HSQC NMR spectrum of **2** in 800 MHz instrument, $CDCl_3$ 12
Figure S11. COSY NMR spectrum of **2** in 800 MHz instrument, $CDCl_3$ 12
Figure S12. HMBC NMR spectrum of **2** in 800 MHz instrument, $CDCl_3$ 13
Figure S13. 1D ROESY NMR spectrum of **2** in 800 MHz instrument, $CDCl_3$ 13
Figure S14. Positive ionization mode HRMS data for **2** 14
Figure S15. ^1H NMR spectrum of **3–4** in 800 MHz instrument, $CDCl_3$ 14
Figure S16. ^{13}C NMR spectrum of **3–4** in 800 MHz instrument, $CDCl_3$ 15
Figure S17. HSQC NMR spectrum of **3–4** in 800 MHz instrument, $CDCl_3$ 15
Figure S18. COSY NMR spectrum of **3–4** in 800 MHz instrument, $CDCl_3$ 16
Figure S19. HMBC NMR spectrum of **3–4** in 800 MHz instrument, $CDCl_3$ 16
Figure S20. 1D ROESY NMR spectrum of **3–4** in 800 MHz instrument, $CDCl_3$ 17
Figure S21. 1D ROESY NMR spectrum of **3–4** in 800 MHz instrument, $CDCl_3$ 17
Figure S23. ^1H NMR spectrum of **5** in 800 MHz instrument, $CDCl_3$ 18
Figure S24. ^{13}C NMR spectrum of **5** in 800 MHz instrument, $CDCl_3$ 19
Figure S25. HSQC NMR spectrum of **5** in 800 MHz instrument, $CDCl_3$ 19
Figure S26. COSY NMR spectrum of **5** in 800 MHz instrument, $CDCl_3$ 20
Figure S27. HMBC NMR spectrum of **5** in 800 MHz instrument, $CDCl_3$ 20
Figure S28. 1D ROESY NMR spectrum of **5** in 800 MHz instrument, $CDCl_3$ 21
Figure S29. 1D ROESY NMR spectrum of **5** in 800 MHz instrument, $CDCl_3$ 21
Figure S30. Positive ionization mode HRMS data for **5** 22
Figure S31. ^1H NMR spectrum of **6** in 800 MHz instrument, $CDCl_3$ 22
Figure S32. Positive ionization mode HRMS data for **6** 23
Figure S33. ^1H NMR spectrum of **7** in 800 MHz instrument, $CDCl_3$ 23
Figure S34. ^{13}C NMR spectrum of **7** in 800 MHz instrument, $CDCl_3$ 24
Figure S35. Positive ionization mode HRMS data for **7** 24
Figure S36. ^1H NMR spectrum of **8** in 800 MHz instrument, $CDCl_3$ 25
Figure S37. ^{13}C NMR spectrum of **8** in 800 MHz instrument, $CDCl_3$ 25
Figure S38. Positive ionization mode HRMS data for **8** 26
Figure S39. ^1H NMR spectrum of **9** in 800 MHz instrument, $CDCl_3$ 26
Figure S40. ^{13}C NMR spectrum of **9** in 800 MHz instrument, $CDCl_3$ 27
Figure S42. ^1H NMR spectrum of **10** in 800 MHz instrument, $CDCl_3$ 28
Figure S43. ^{13}C NMR spectrum of **10** in 800 MHz instrument, $CDCl_3$ 28
Figure S45. ^1H NMR spectrum of **11** and **12** in 800 MHz instrument, pyridine-d*5* 29
Figure S46. ^{13}C NMR spectrum of **11** and **12** in 800 MHz instrument, pyridine-d*5* 30
Figure S47. Positive ionization mode HRMS data for **11–12** 30
Figure S48. ^1H NMR spectrum of **13** in 800 MHz instrument, $CDCl_3$ 31
Figure S49. ^{13}C NMR spectrum of **13** in 800 MHz instrument, $CDCl_3$ 31

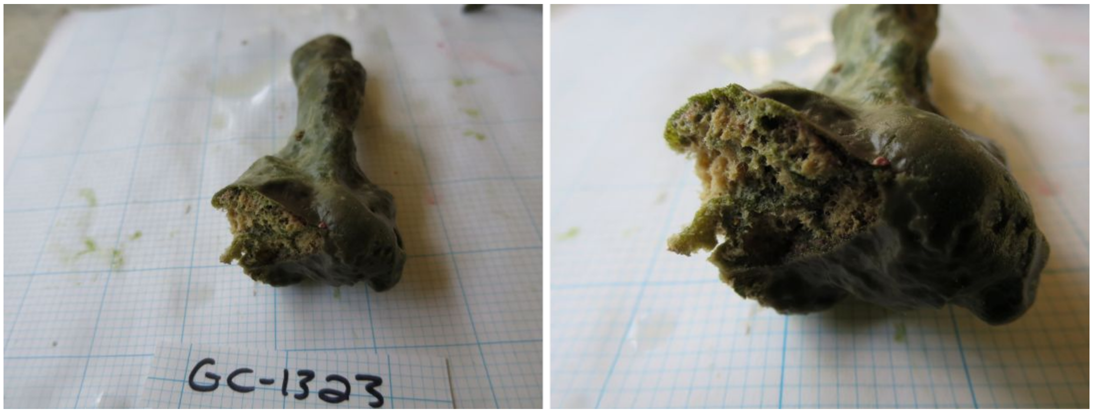

Figure S1. Collection photo for *Petrosia* sp. marine sponge .

Characterization data for distrongylophorine (**6**): ^{1}H NMR (800 MHz, $CDCl_3$): δ_H 6.81 (d, J = 2.6 Hz, 1H), 6.80 (d, J = 8.9 Hz, 1H), 6.60 (d, J = 8.9 Hz, 1H), 6.57 (d, J = 8.9 Hz, 1H), 6.41 (d, J = 8.9, 2.6 Hz, 1H), 2.62 (m, 2H), 2.32 (dd, J = 17.5, 4.5 Hz, 1H), 2.20 (m, 1H), 2.16 (br dd, J = 13.7, 13.7 Hz, 2H), 2.01 (br dd, J = 12.8, 12.8 Hz, 1H), 2.01 (m, 1H), 1.92 (m, 1H), 1.87 (m, 1H), 1.85–1.77 (m, 5H), 1.77–1.68 (m, 3H), 1.68–1.61 (m, 3H), 1.59 (m, 1H), 1.47 (m, 3H), 1.43 (dd, J = 12.8, 4.4 Hz, 1H), 1.34 (m, 1H), 1.29 (m, 1H), 1.26 (s, 3H), 1.20 (s, 3H), 1.15 (s, 3H), 1.12 (s, 3H), 1.10 (d, J = 13.0 Hz, 1H), 1.05–1.00 (m, 4H), 0.98 (d, J = 12.2 Hz, 1H), 0.94 (s, 3H), 0.92 (m, 1H), 0.88 (m, 1H), 0.88 (d, J = 12.1 Hz, 1H), 0.82 (s, 3H), 0.80 (s, 3H), 0.76 (s, 3H), 0.72 (br dd, J = 12.8, 12.8 Hz, 1H); HRMS *m/z* $[M + H]^+$ calcd for $C_{52}H_{71}O_8^+$ 823.5144, found 823.5142.[1]

Characterization data for strongylophorine-1 (**7**): ^{1}H NMR (800 MHz, $CDCl_3$): δ_H 6.68–6.65 (m, 2H), 6.62 (d, J = 2.8 Hz, 1H), 3.74 (s, 3H), 2.61 (d, J = 9.1 Hz, 2H), 2.15 (br d, J = 13.5 Hz, 1H), 2.03 (m, 1H), 2.02–1.73 (m, 6H), 1.66–1.60 (m, 2H), 1.47 (br d, J = 14.3 Hz, 1H), 1.37–1.28 (m, 2H), 1.25 (s, 3H), 1.17 (s, 3H), 1.12–1.09 (m, 1H), 1.06–0.94 (m, 3H), 0.92 (s, 3H), 0.80 (s, 3H); ^{13}C NMR (201 MHz, $CDCl_3$): 183.3, 153.1, 147.3, 123.1, 117.6, 114.5, 113.2, 76.6, 60.2, 57.1, 55.8, 52.5, 43.9, 41.3, 40.9, 40.3, 38.1, 38.0, 37.1, 28.9, 22.8, 20.7, 19.7, 19.2, 19.0, 15.7, 14.3; HRMS *m/z* $[M + H]^+$ calcd for $C_{27}H_{39}O_4^+$ 427.2843, found 427.2842.[2]

Characterization data for strongylophorine-3 (**8**): ^{1}H NMR (800 MHz, $CDCl_3$): δ_H 6.61 (d, J = 9.3 Hz, 1H), 6.57 (m, 2H), 2.56 (d, J = 9.0 Hz, 2H), 2.15 (d, J = 13.2 Hz, 1H), 2.04 (m, 1H), 1.99 (m, 1H), 1.90 (m, 1H), 1.83–1.78 (m, 3H), 1.74 (d, 13.6 Hz, 1H), 1.64–1.61 (m, 2H), 1.47 (d, J = 14.3 Hz, 1H), 1.32 (m, 1H), 1.24 (s, 3H), 1.16 (s, 3H), 1.10 (m, 1H), 1.03 (m, 1H), 1.01 (m, 1H), 0.96 (d, J = 12.2 Hz, 1H), 0.93 (m, 1H), 0.91 (s, 3H), 0.79 (s, 3H); 183.7, 148.9, 147.1, 123.2, 117.6, 115.9, 114.4, 76.5, 60.2, 57.1, 52.5, 43.9, 41.2, 40.9, 40.2, 38.1, 38.0, 37.1, 28.9, 22.6, 20.7, 19.7, 19.2, 18.9, 15.7, 14.3; HRMS *m/z* $[M + H]^+$ calcd for $C_{26}H_{37}O_4^+$ 413.2687, found 413.2685.[2]

Characterization data for strongylophorine-4 (**9**): ^{1}H NMR (800 MHz, $CDCl_3$): δ_H 9.80 (s, 1H), 6.62 (d, J = 8.5 Hz, 1H), 6.56 (d, J = 8.5 Hz, 1H), 6.55 (s, 1H), 2.57 (m, 2H), 2.12 (d, J = 13.4 Hz, 1H), 2.03 (d, J = 12.2 Hz, 1H), 1.87 (m, 2H), 1.83–1.73 (m, 3H), 1.70–1.62 (m, 3H), 1.49 (m, 1H), 1.33 (m, 1H), 1.21 (m, 2H), 1.16 (s, 3H), 1.08–1.04 (m, 2H), 1.01 (s, 3H), 0.92 (m, 1H), 0.90 (s, 3H), 0.73 (s, 3H); ^{13}C NMR (201 MHz, $CDCl_3$): 205.9, 149.0, 147.1, 123.1, 117.6, 115.9, 114.4, 76.4, 59.7, 56.9, 52.5, 48.5, 41.2, 40.9, 39.4, 37.9, 37.1, 34.6, 24.3, 22.6, 20.7, 19.0, 18.5, 18.0, 16.0, 15.3; HRMS *m/z* $[M–H]^-$ calcd for $C_{26}H_{35}O_3^-$ 395.2591, found 395.2596.[1]

Characterization data for strongylophorine-9 (**10**): ^{1}H NMR (800 MHz, $CDCl_3$): δ_H 6.68–6.65 (m, 2H), 6.61 (d, J = 2.0 Hz, 1H), 4.78 (dd, J = 12.3, 2.2 Hz, 1H), 4.02 (d, J = 12.3 Hz, 1H), 3.74 (s, 3H), 2.65–2.59 (m, 2H), 2.17 (br d, J = 13.3 Hz, 1H), 2.09 (m, 1H), 1.89–1.81 (m, 4H), 1.78–1.63 (m, 4H), 1.53–1.49 (m, 1H), 1.36–1.28 (m, 2H), 1.28–1.22 (m, 2H), 1.21 (s, 3H), 1.17 (s, 3H), 1.16–1.06 (m, 2H), 1.01 (s, 3H); ^{13}C NMR (201 MHz, $CDCl_3$): 176.7, 153.2, 147.0, 122.4, 117.6, 114.4, 113.4, 76.1, 73.6, 55.8, 55.4, 52.6, 50.6, 43.3, 41.5, 40.3, 40.3, 38.3, 36.8, 36.6, 23.3, 22.6, 21.1, 20.9, 20.5, 18.8, 15.9; HRMS *m/z* $[M + H]^+$ calcd for $C_{27}H_{37}O_4^+$ 425.2687, found 425.2685.[2]

Characterization data for strongylophorine-15 (**11**) and strongylophorine-16 (**12**): ^{1}H NMR (800 MHz, pyridine-d_5): δ_H 7.08–7.06 (m, 4H), 7.02–7.00 (m, 2H), 5.23 (d, J = 2.3 Hz, 1H), 5.11 (d, J = 3.4 Hz, 1H), 4.64 (dd, J = 11.3, 2.6 Hz, 1H), 4.23 (dd, J = 11.3, 2.6 Hz, 1H), 3.70 (dd, J = 11.6, 1.0 Hz, 1H), 3.44 (d, J = 11.3 Hz, 1H), 2.86 (m, 1H), 2.69–2.59 (m, 4H), 2.59–2.53 (m, 3H), 2.21 (m, 2H), 2.12 (m, 2H), 1.80–1.75 (m, 5H), 1.73–1.66 (m, 5H), 1.62–1.56 (m, 4H), 1.37 (m, 1H), 1.23 (m, 1H), 1,20 (s, 3H), 1.19 (s, 3H), 1.16 (m, 2H), 1.14 (s, 3H), 1.07 (s, 3H), 1.01 (m, 2H), 0.97 (m, 2H), 0.92 (m, 2H), 0.89 (s, 3H), 0.87 (m, 2H), 0.78 (s, 3H); ^{13}C NMR (201 MHz, pyridine-d_5): 152.5, 152.4, 147.1, 147.1, 123.5, 123.5, 118.3, 118.3, 117.2, 117.2, 115.7, 115.7, 99.5, 99.2, 76.7, 76.6, 68.3, 62.4, 57.9, 57.3, 53.3, 53.3, 51.9, 49.4, 42.5, 42.5, 41.1, 40.9, 40.8, 39.7, 39.2, 38.2, 38.0, 37.8, 37.5, 36.9, 36.7, 35.5, 24.6, 24.4, 23.3, 23,3, 23.3, 23.1, 21.4, 21.3, 20.8, 20.8, 19.4, 18.4, 16.1, 16.0; HRMS *m/z* $[M + H]^+$ calcd for $C_{26}H_{37}O_4^+$ 413.2687, found 413.2685.[3]

Table S1. NMR spectral data for 20-*O*-methyl strongylophorine-15 (**3**) in 800 MHz instrument, $CDCl_3$, isolated as a mixture with 20-*O*-methyl strongylophorine-16 (**4**).

position	δ_C	$\delta_{H(mult, J\ Hz)}$	COSY	HMBC
1a	40.2 or 40.3 (CH_2)	1.11 m	H-2a, H-2b	C-2, C-9, C-10, C-24
1b		2.24 m	H-2a, H-2b	C-2, C-5, C-10, C-24
2a	22.1 or 22.3 (CH_2)	1.52 m	H-1a, H-1b, H-3a, H-3b	C-1, C-3
2b		2.33 m	H-1a, H-1b, H-3a, H-3b	C-1, C-3
3a	34.3 (CH_2)	1.17 m	H-2a, H-2b, H-3b	C-2, C-4, C-5, C-25, C-26
3b		1.98 m	H-2a, H-2b, H-3a	C-1, C-2, C-4, C-5, C-25, C-26
4	37.1 (C)			
5	51.4 (CH)	1.21 m	H-6a, H-6b	C-3, C-6, C-24, C-26
6a	17.7 (CH_2)	1.64 m	H-5, H-7a, H-7b	C-5, C-7, C-8, C-10
6b		1.64 m	H-5, H-7a, H-7b	C-5, C-7, C-8, C-10
7a	38.8 (CH_2)	1.00 m	H-6a, H-6b, H-7b	
7b		1.82 ddd (12.6, 3.4, 3.4)	H-6a, H-6b, H-7a	C-5, C-6, C-8, C-9, C-23
8	36.3 or 36.5 (C)			
9	56.7 (CH)	1.03 m	H-11a, H-11b	C-1, C-8, C-10, C-11, C-12, C-14, C-23, C-24
10	37.4 or 37.7 (C)			
11a	18.9 or 19.0 (CH_2)	1.22 m	H-9, H-12a, H-12b	C-8, C-9, C-12, C-13
11b		1.87 m	H-9, H-12a, H-12b	C-8, C-9, C-10, C-12, C-13
12a	41.7 (CH_2)	1.64 m	H-11a, H-11b, H-12b	C-9, C-13, C-22
12b		2.05 ddd (12.6, 2.8, 2.8)	H-11a, H-11b, H-12a	C-9, C-11, C-13, C-14, C-22
13	76.4 or 76.5 (C)			
14	52.6 (CH)	1.63 m	H-15a, H-15b	C-8, C-9, C-13, C-15, C-16, C-22, C-23
15a	22.9 (CH_2)	2.6 m	H-14	C-8, C-13, C-14, C-16, C-21
15b		2.6 m	H-14	C-8, C-13, C-14, C-16, C-21
16	122.9 (C)			
17	147.2 (C)			
18	117.6 (CH)	6.67 d (8.8)		C-16, C-17, C-20, C-21
19	113.3 (CH)	6.65 m		C-17, C-20, C-21
20	153.1 (C)			
21	114.4 or 114.5 (CH)	6.61 d (2.8)		C-17, C-19, C-20
22	20.9 (CH_3)	1.14 s or 1.15 s		C-12, C-13, C-14
23	15.7 (CH_3)	0.89 s		C-7, C-8, C-9, C-14
24a	68.3 (CH_2)	3.59 d (11.7)	H-24b	C-1, C-5, C-10, C-26
24b		4.15 dd (11.7, 2.6)	H-24a	C-1, C-5, C-9, C-26
25	23.3 (CH_3)	0.86 s		C-3, C-4, C-5
26	99.0 (CH)	4.82 d (4.2)	26-OH	C-3, C-4, C-25
	55.9 (OCH_3)	3.74 s		C-20
26-OH		2.51 d (5.8)	H-26	C-26

Table S2. NMR spectral data for 20-*O*-methyl strongylophorine-16 (**4**) in 800 MHz instrument, $CDCl_3$, isolated as a mixture with 20-*O*-methyl strongylophorine-15 (**3**).

position	δ_C	$\delta_{H (mult, J Hz)}$	COSY	HMBC
1a	40.2 or 40.3 (CH_2)	1.11 m	H-2a, H-2b	C-2, C-9, C-10, C-24
1b		2.24 m	H-2a, H-2b	C-2, C-5, C-10, C-24
2a	22.1 or 22.3 (CH_2)	1.52 m	H-1a, H-1b, H-3a	C-1, C-3
2b		2.23 m	H-1a, H-3a	C-1, C-3
3a	40.5 (CH_2)	1.33 m	H-2a, H-2b	C-2, C-4, C-5, C-25, C-26
3b		1.52 m	H-2a, H-2b	C-1, C-2, C-4, C-5, C-25, C-26
4	36.7 (C)			
5	48.5 (CH)	1.05 m	H-6a, H-6b	C-1, C-9, C-24, C-26
6a	20.0 (CH_2)	1.67 m	H-5, 6b, H-7a, H-7b	C-4, C-5, C-10
6b		2.18 m	H-5, 6a, H-7a, H-7b	C-4, C-5, C-7
7a	39.2 (CH_2)	0.90 m	H-6a, H-6b, 7b	
7b		1.78 ddd (13.0, 3.4, 3.4)	H-6a, H-6b, 7a	C-5, C-6, C-8, C-9, C-23
8	36.3 or 36.5 (C)			
9	57.4 (CH)	0.98 m	H-11a, H-11b	C-1, C-8, C10, C-11, C-12, C-23
10	37.4 or 37.7 (C)			
11a	18.9 or 19.0 (CH_2)	1.24 m	H-9, H-12a, H-12b	C-8, C-9, C-12, C-13
11b		1.87 m	H-9, H-12a, H-12b	C-8, C-9, C-10, C-12, C-13
12a	41.7 (CH_2)	1.64 m	H-11a, H-11b	C-9, C-13, C-22
12b		2.05 ddd (12.6, 2.8, 2.8)	H-11a, H-11b	C-9, C-11, C-13, C-14, C-22
13	76.4 or 76.5 (C)			
14	52.6 (CH)	1.63 m	H-15a, H-15b	C-8, C-9, C-13, C-15, C-16, C-22, C-23
15a	22.9 (CH_2)	2.6 m	H-14	C-8, C-13, C-14, C-16, C-17, C-18, C-21
15b		2.6 m	H-14	C-8, C-13, C-14, C-16, C-17, C-18, C-21
16	122.7 (C)			
17	147.2 (C)			
18	117.6 (CH)	6.67 d (8.8)		C-16, C-17, C-20, C-21
19	113.3 (CH)	6.65 m		C-17, C-20, C-21
20	153.1 (C)			
21	114.4 or 114.5 (CH)	6.61 d (2.8)		C-15, C-17, C-19, C-20
22	20.9 (CH_3)	1.14 s or 1.15 s		C-12, C-13, C-14
23	15.9 (CH_3)	0.91 s		C-7, C-8, C-9, C-14
24a	62.4 (CH_2)	3.30 d (11.2)	H-24b	C-1, C-5, C-10, C-26
24b		4.31 dd (11.3, 2.5)	H-24a	C-1, C-5, C-10, C-26
25	23.5 (CH_3)	0.94 s		C-3, C-4, C-5, C-26
26	99.7 (CH)	4.76 s		C-3, C-5, C-24
	55.9 (OCH_3)	3.74 s		C-20

Table S3: NMR spectral data for distrongylophorine A (**5**) in 800 MHz instrument, $CDCl_3$

position	δ_C	$\delta_{H(mult, J\ Hz)}$	COSY	HMBC	1D ROESY
1a	39.4 (CH_2)	0.92 m	H-2a, H-2b		
1b		1.76 m	H-2a		
2a	18.5 (CH_2)	1.49 m	H-1a, H-1b, H-3a, H-3b		
2b		1.66 m	H-1a, H-3a, H-3b		
3a	34.6 (CH_2)	1.03 m	H-2a, H-2b, H-3b		
3b		2.11 m	H-2a, H-2b, H-3a		
4	48.5 (C)				
5	56.9 or 57.0 (CH)	1.18 m	H-6a, H-6b	C-4, C-10, C-24, C-26	
6a	17.9 or 18.0 (CH_2)	1.79 m	H-5, H-7a		
6b		1.86 m	H-5		
7a	40.7 or 40.9 (CH_2)	1.03 m	H-6a		
7b		1.86 m			
8	37.0 or 37.1 (C)				
9	59.7 (CH)	0.98 m	H-11a		
10	37.8 or 37.9 (C)				
11a	18.9 or 19.0 (CH_2)	1.33 m	H-9, H-12a, H-12b		
11b		1.74 m	H-12b		
12a	41.2 (CH_2)	1.64 m	H-11a	C-9, C-13, C-22	
12b		2.04 m	H-11a, H-11b	C-9, C-13, C-14, C-22	
13	76.2 or 76.8 (C)				
14	52.4 (CH)	1.63 m	H-15a, H-15b	C-8, C-9, C-12, C-13, C-15, C-22, C-23	
15a	22.7 (CH_2)	2.56 d (9.1)	H-14	C-8, C-13, C-14, C-16, C-17, C-21	
15b		2.56 d (9.1)	H-14	C-8, C-13, C-14, C-16, C-17, C-21	
16	123.5 (C)				
17	148.5 (C)				
18	118.1 (CH)	6.67 d (8.8)		C-16, C-17, C-20	
19	114.0 (CH)	6.64 dd (8.8, 3.0)		C-17, C-21	
20	150.5 (C)				
21	115.4 (CH)	6.58 d (3.0)		C-15, C-17, C-19	
22	20.7 (CH_3)	1.16 s		C-12, C-13, C-14	H-23
23	16.0 (CH_3)	0.89 s		C-7, C-8, C-9, C-14	H-22, H-24
24	15.3 (CH_3)	0.73 s		C-1, C-5, C-9, C-10	H-23, H-26
25	24.3 (CH_3)	1.01 s		C-3, C-4, C-5, C-26	H-26
26	205.8 (CH)	9.79 s		C-3, C-4	H-24, H-25
1a′	39.4 (CH_2)	0.92 m	H-2a′, H-2b′		
1b′		1.76 m	H-2a′		
2a′	18.5 (CH_2)	1.49 m	H-1a′, H-1b′, H-3a′, H-3b′		
2b′		1.66 m	H-1a′, H-3a′, H-3b′		
3a′	34.6 (CH_2)	1.03 m	H-2a′, H-2b′, 3b′		
3b′		2.11 dd (12.5, 12.5)	H-2a′, H-2b′, 3a′		
4′	48.5 (C)				
5′	56.9 or 57.0 (CH)	1.18 m	H-6a′, H-6b′	C-4′, C-10′, C-24′, C-26′	
6a′	17.9 or 18.0 (CH_2)	1.79 m	H-5′		
6b′		1.86 m	H-5′		
7a′	40.7 or 40.9 (CH_2)	0.91 m	H-6a′		
7b′		1.69 m			
8′	37.0 or 37.1 (C)				
9′	59.7 (CH)	0.98 m	H-11a′		
10′	37.8 or 37.9 (C)				
11a′	18.9 or 19.0 (CH_2)	1.33 m	H-9′, H-12a′, H12-b′		
11b′		1.74 m	H-12b′		
12a′	41.2 (CH_2)	1.64 m	H-11a′	C-9′, C-13′, C-22′	
12b′		2.04 m	H-11a′, H-11b′	C-9′, C-13′, C-14′, C-22′	
13′	76.2 or 76.8 (C)				
14′	51.8 (CH)	1.54 m	H-15a′, H15b′	C-8′, C-13′, C-22′, C-23′	
15a′	17.8 (CH_2)	2.51 dd (17.1, 5.0)	H-14′	C-13′, C-14′, C-16′, C-17′, C-21′	
15b′		2.17 dd (17.1, 13.0)	H-14′	C-8′, C-14′, C-16′, C-17′, C-21′	
16′	116.8 (C)				
17′	139.9 (C)				
18′	142.1 (C)				
19′	114.4 (CH)	6.80 d (8.9)	H-20′	C-17′, C-18′, C-21′	
20′	114.0 (CH)	6.58 d (8.9)	H-19′	C-16′, C-18′, C-19′, C-21′	
21′	147.4 (C)				
22′	20.6 (CH_3)	1.12 s		C-12′, C-13′, C-14′	H-23′
23′	15.9 (CH_3)	0.79 s		C-7′, C-8′, C-9′, C-14′	H-15b′, H-22′, H-24′

24′	15.3 (CH_3)	0.69 s		C-1′, C-5′, C-9′, C-10′	H-23′, H-26′
25′	24.3 (CH_3)	0.99 s		C-3′, C-4′, C-5′, C-26′	H-26′
26′	205.8 (CH)	9.76 s		C-3′, C-4′	H-24′, H-25′
18′-OH		4.84 s		C-17′, C-18′, C-19′	

Figure S2. ^{1}H NMR spectrum of **1** in 800 MHz instrument, $CDCl_3$

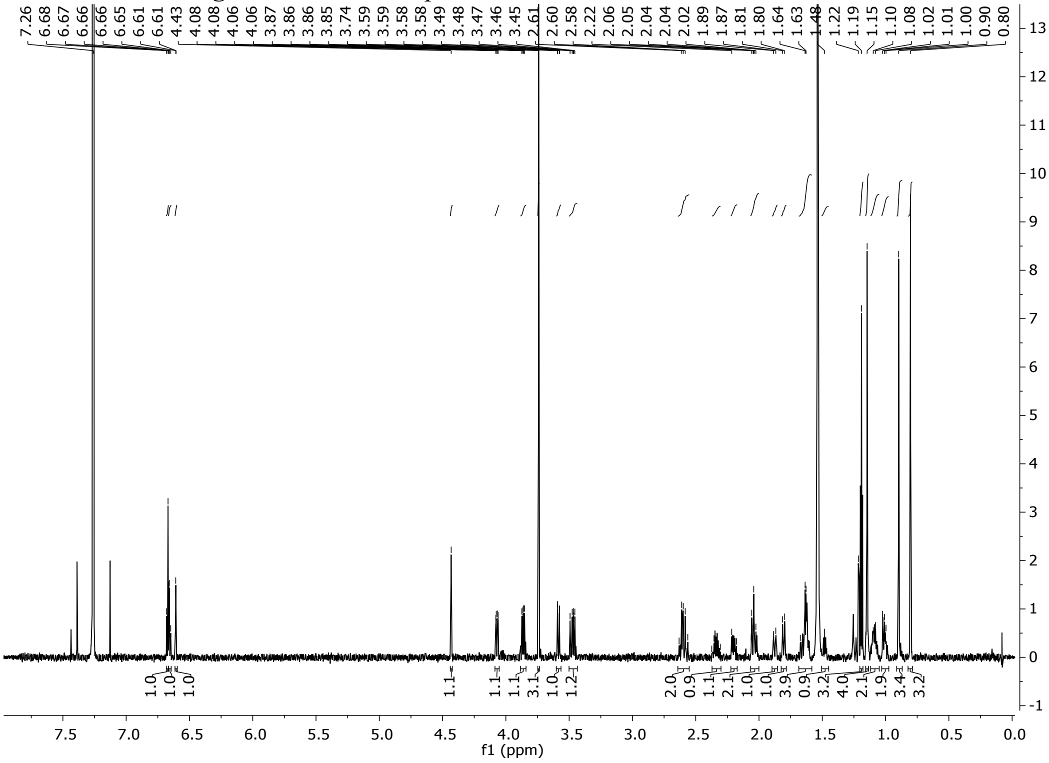

Figure S3. HSQC NMR spectrum of **1** in 800 MHz instrument, $CDCl_3$

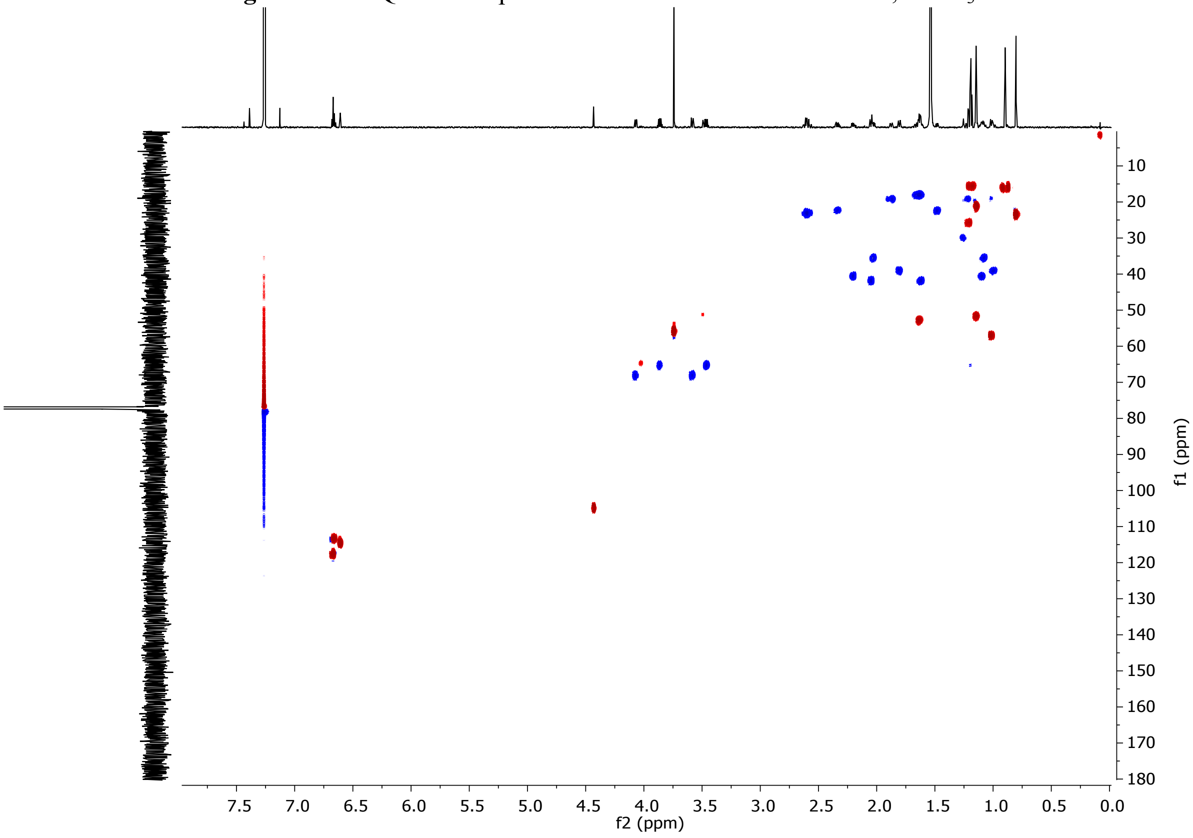

Figure S4. COSY NMR spectrum of **1** in 800 MHz instrument, $CDCl_3$

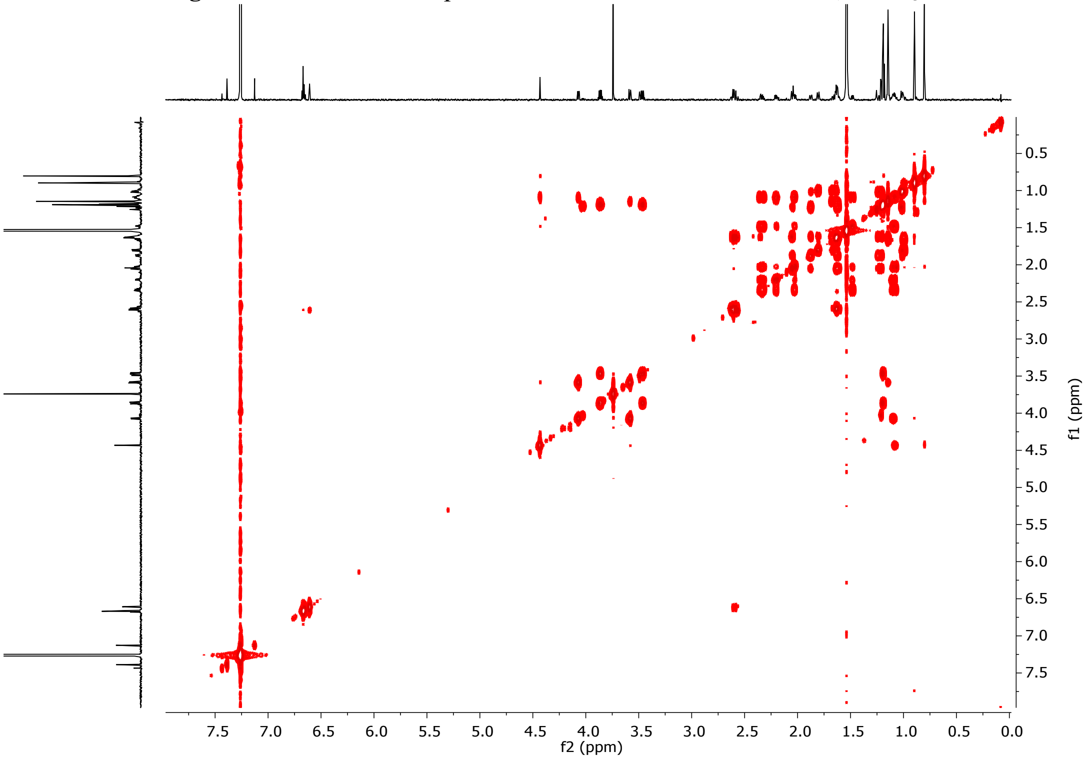

Figure S5. HMBC NMR spectrum of **1** in 800 MHz instrument, $CDCl_3$

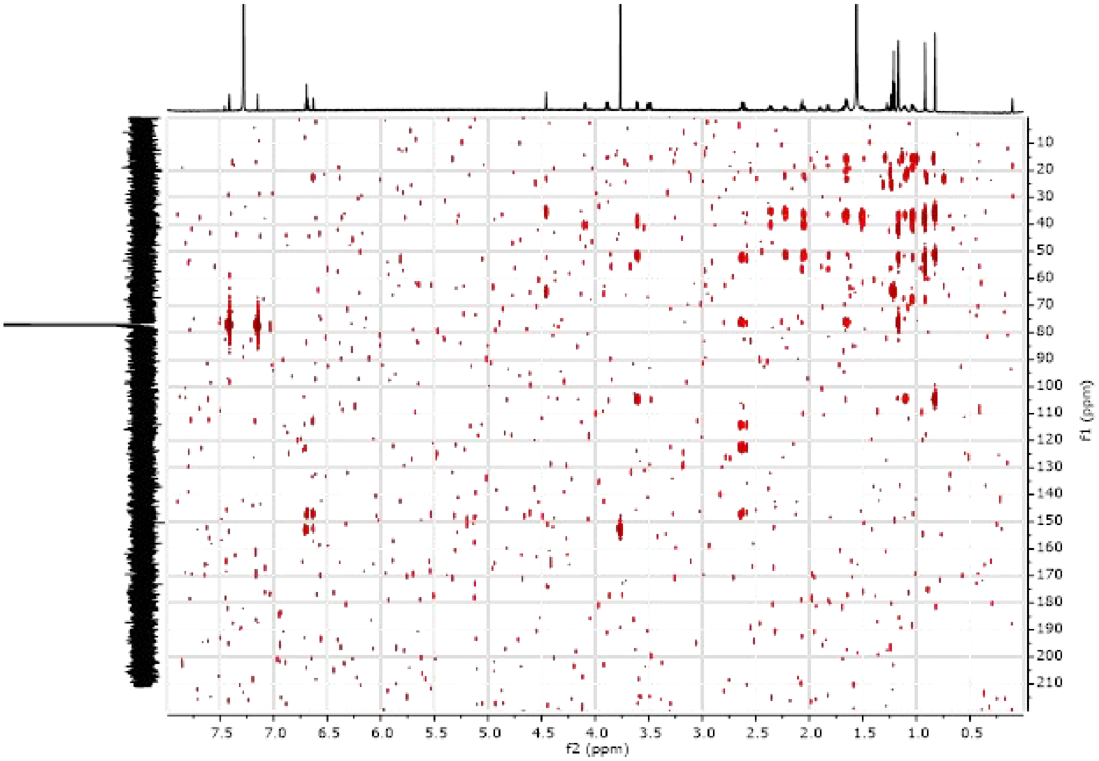

Figure S6. 1D ROESY NMR spectrum of **1** in 800 MHz instrument, $CDCl_3$

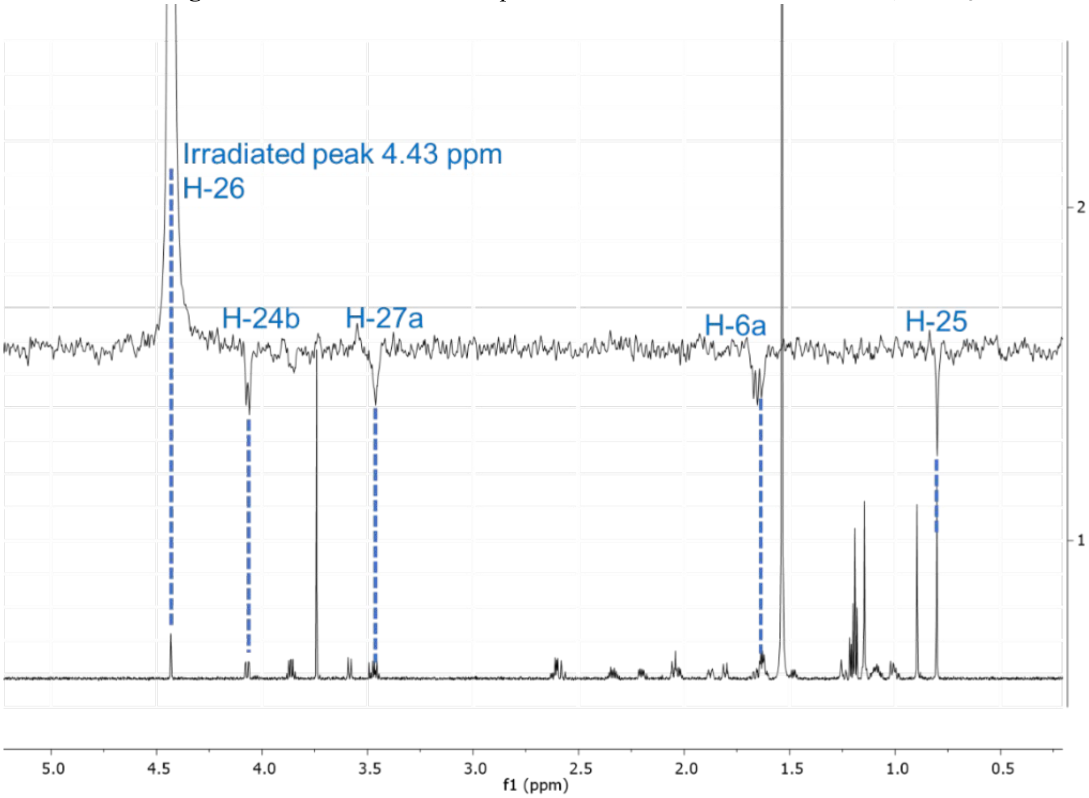

Figure S7. Positive ionization mode HRMS data for **1**

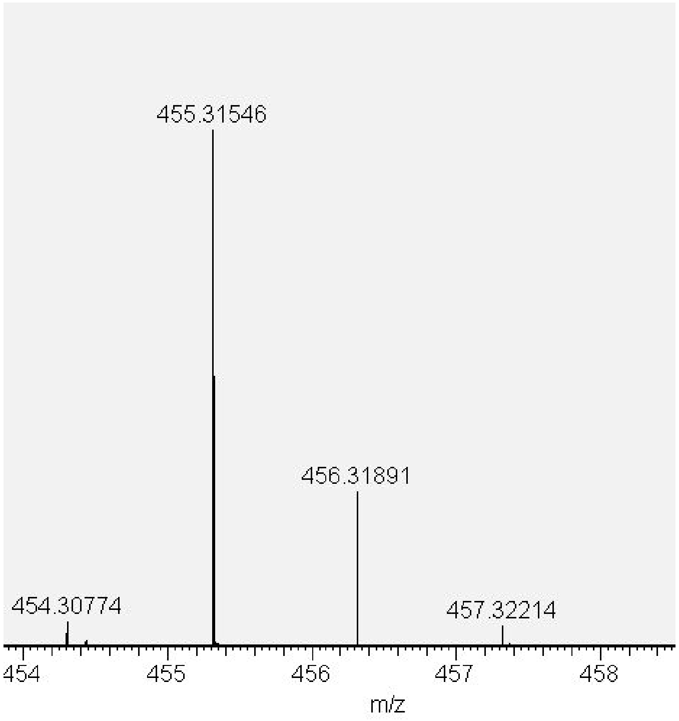

Figure S8. ^{1}H NMR spectrum of **2** in 800 MHz instrument, $CDCl_3$

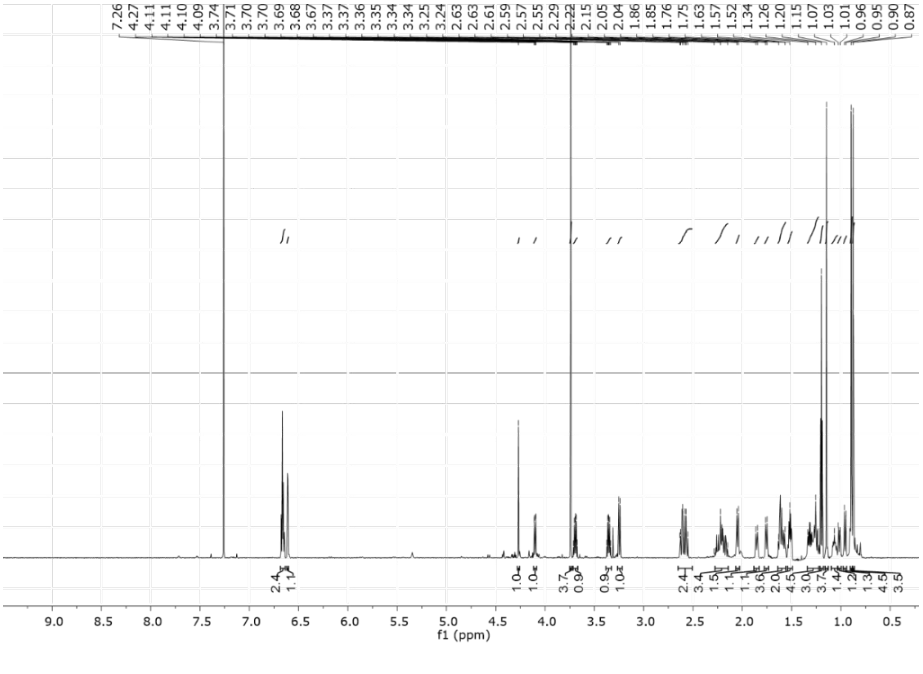

Figure S9. ^{13}C NMR spectrum of **2** in 800 MHz instrument, $CDCl_3$

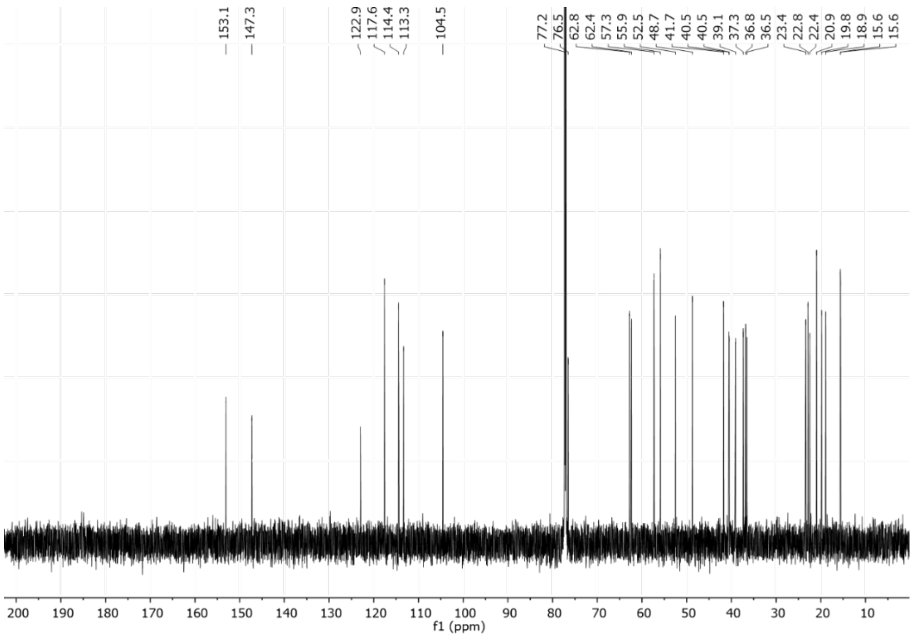

Figure S10. HSQC NMR spectrum of **2** in 800 MHz instrument, $CDCl_3$

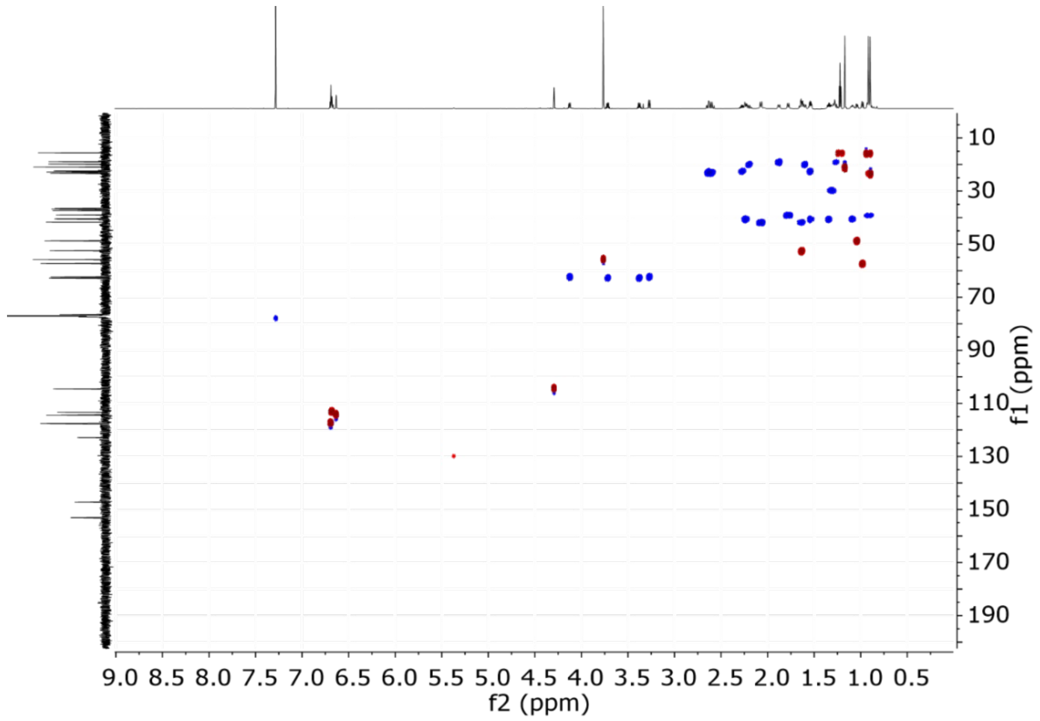

Figure S11. COSY NMR spectrum of **2** in 800 MHz instrument, $CDCl_3$

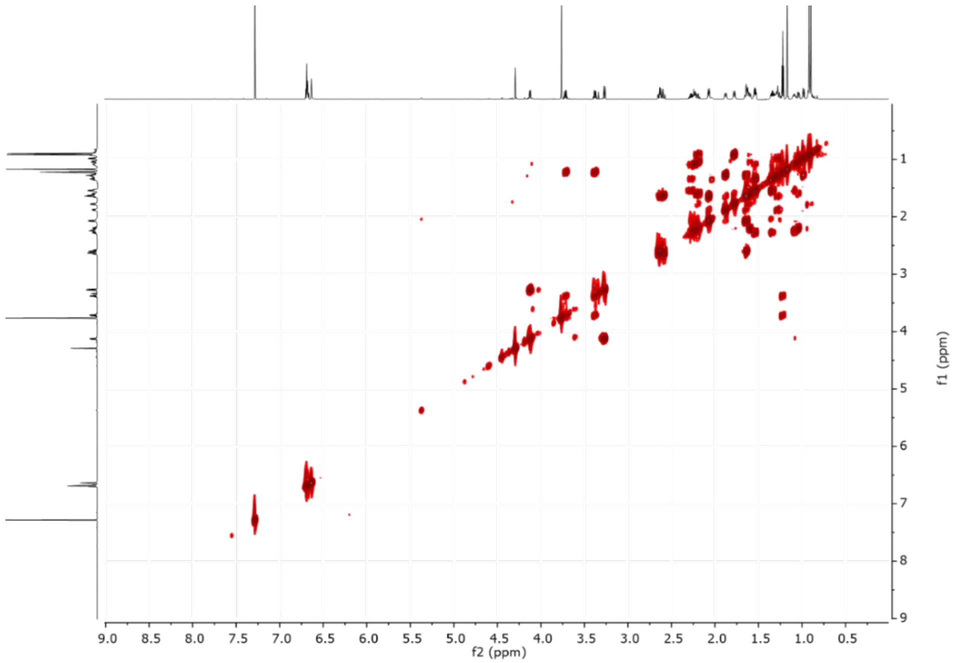

Figure S12. HMBC NMR spectrum of **2** in 800 MHz instrument, $CDCl_3$

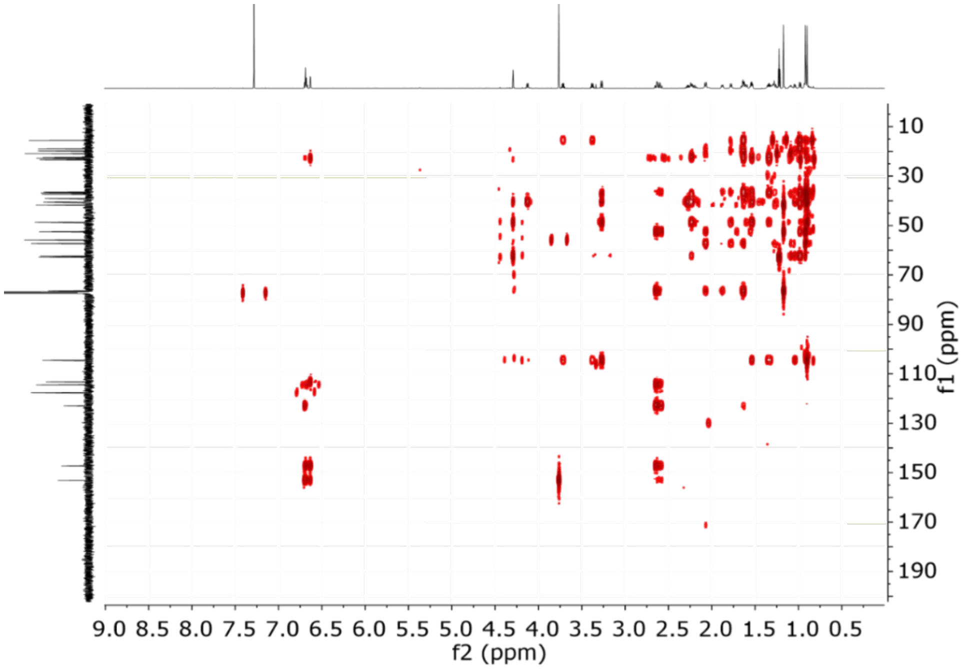

Figure S13. 1D ROESY NMR spectrum of **2** in 800 MHz instrument, $CDCl_3$

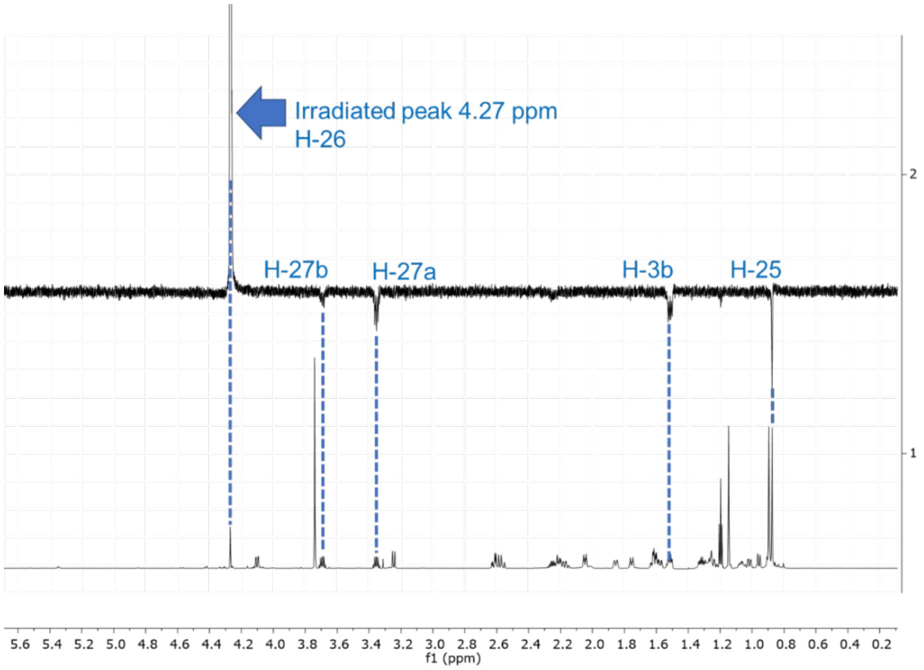

Figure S14. Positive ionization mode HRMS data for **2**

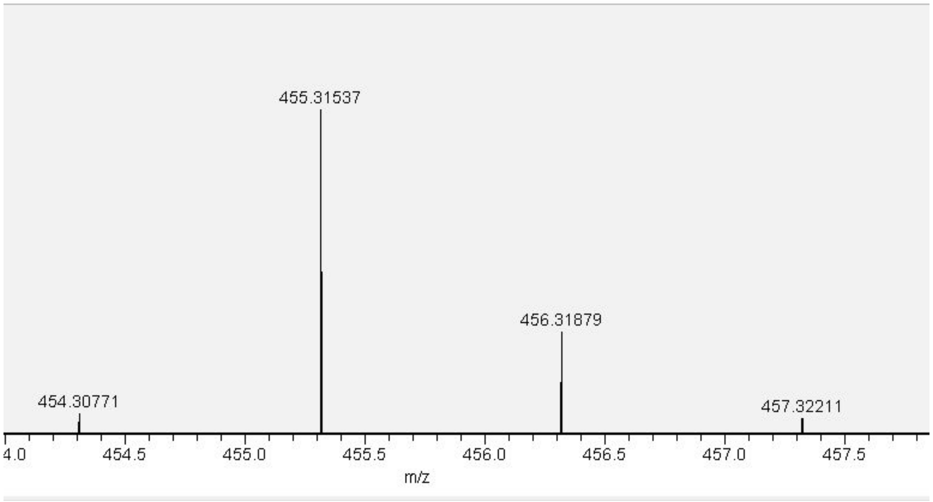

Figure S15. ^{1}H NMR spectrum of **3–4** in 800 MHz instrument, $CDCl_3$

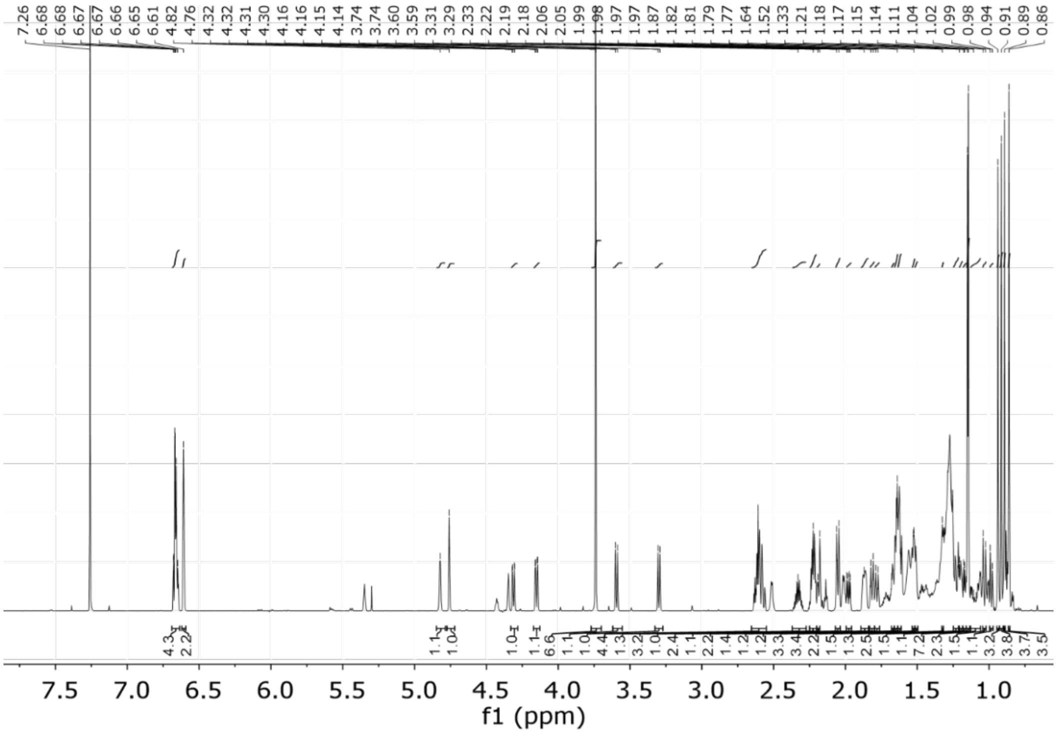

Figure S16. ^{13}C NMR spectrum of **3–4** in 800 MHz instrument, $CDCl_3$

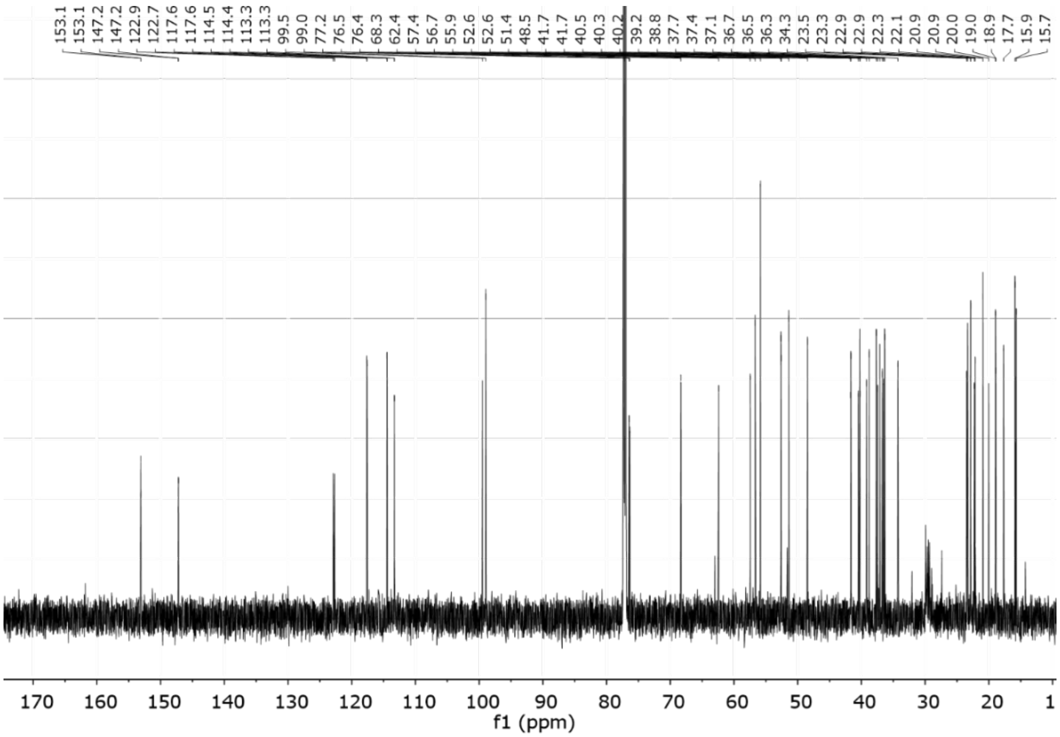

Figure S17. HSQC NMR spectrum of **3–4** in 800 MHz instrument, $CDCl_3$

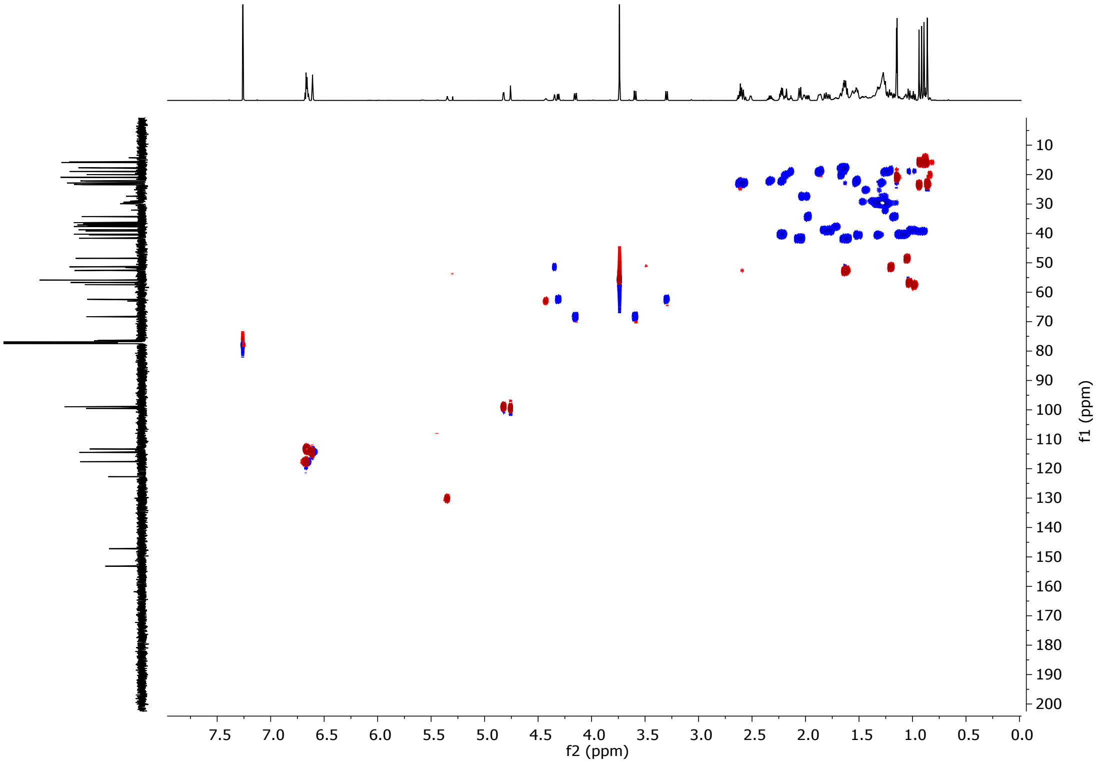

Figure S18. COSY NMR spectrum of **3–4** in 800 MHz instrument, $CDCl_3$

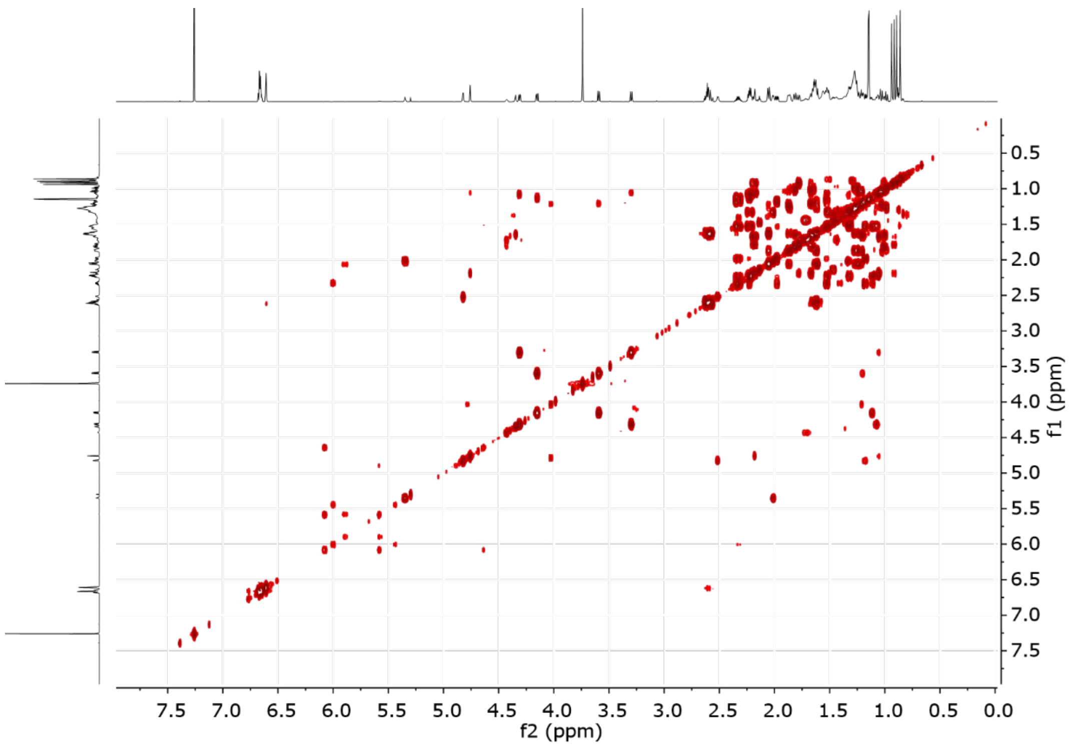

Figure S19. HMBC NMR spectrum of **3–4** in 800 MHz instrument, $CDCl_3$

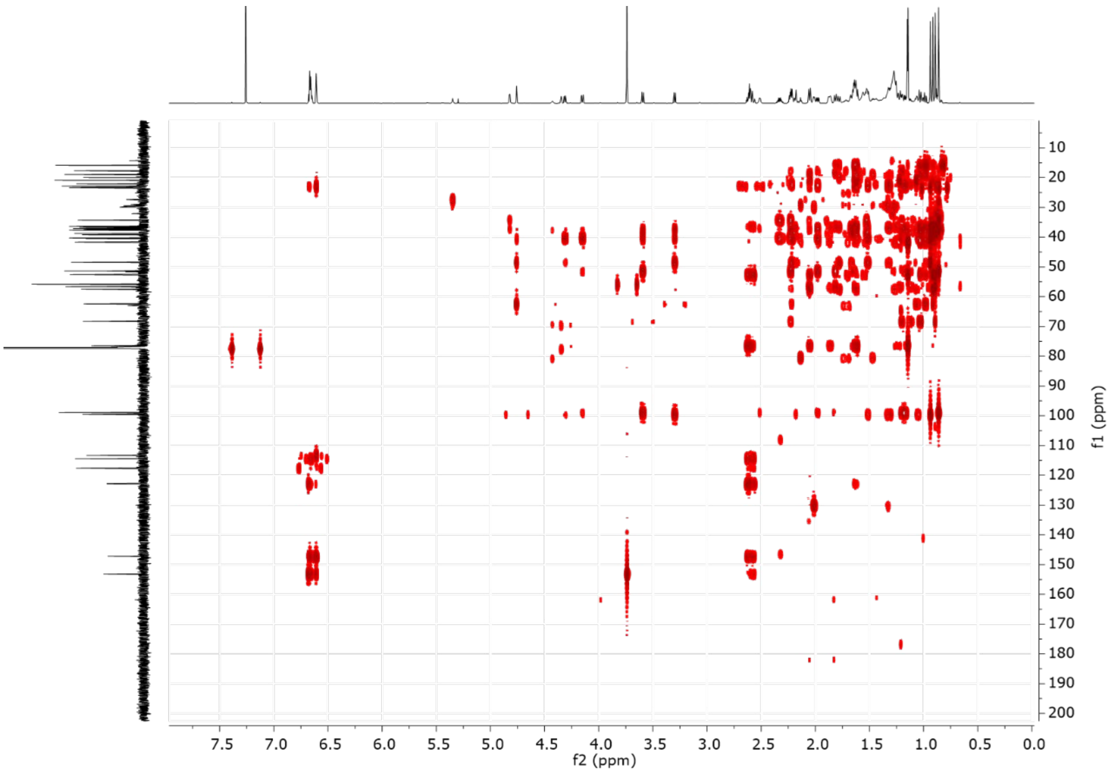

Figure S20. 1D ROESY NMR spectrum of **3–4** in 800 MHz instrument, $CDCl_3$

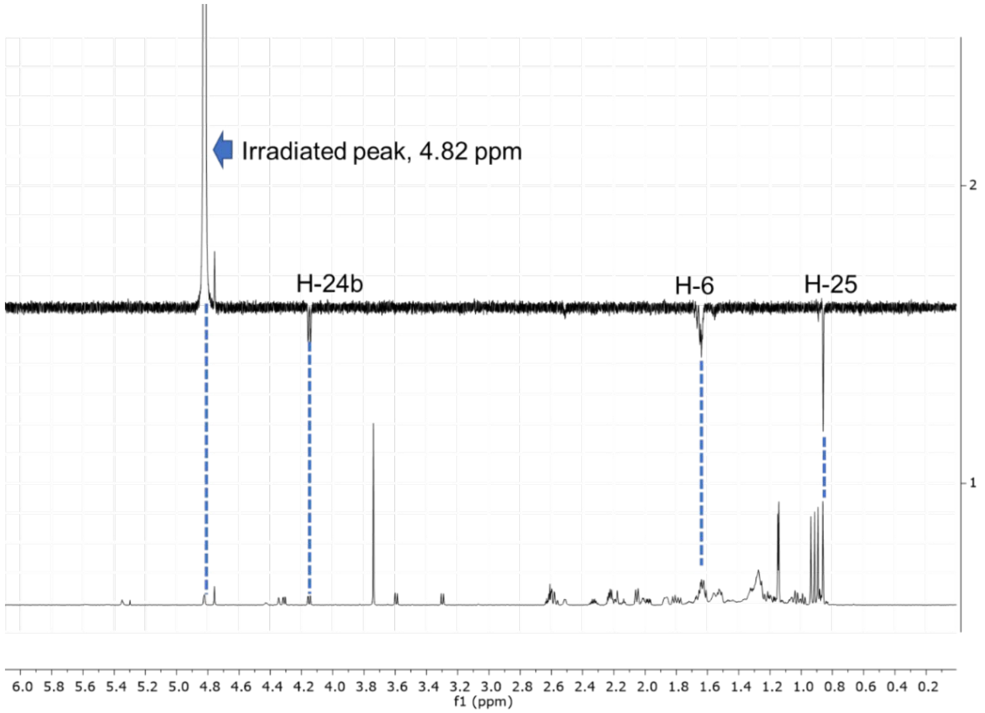

Figure S21. 1D ROESY NMR spectrum of **3–4** in 800 MHz instrument, $CDCl_3$

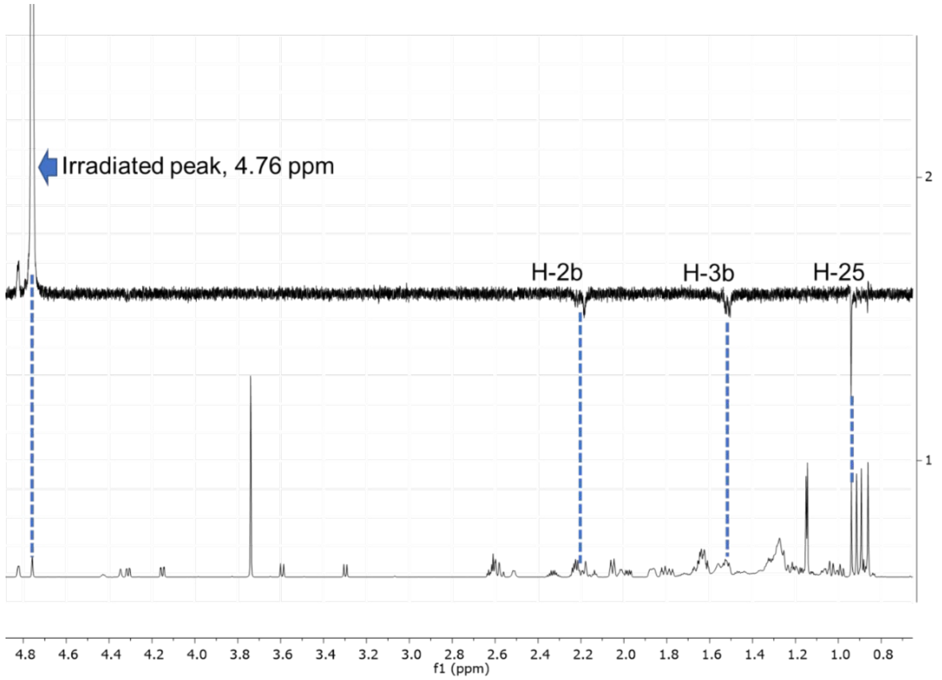

Figure S22. Positive ionization mode HRMS data for **3–4**

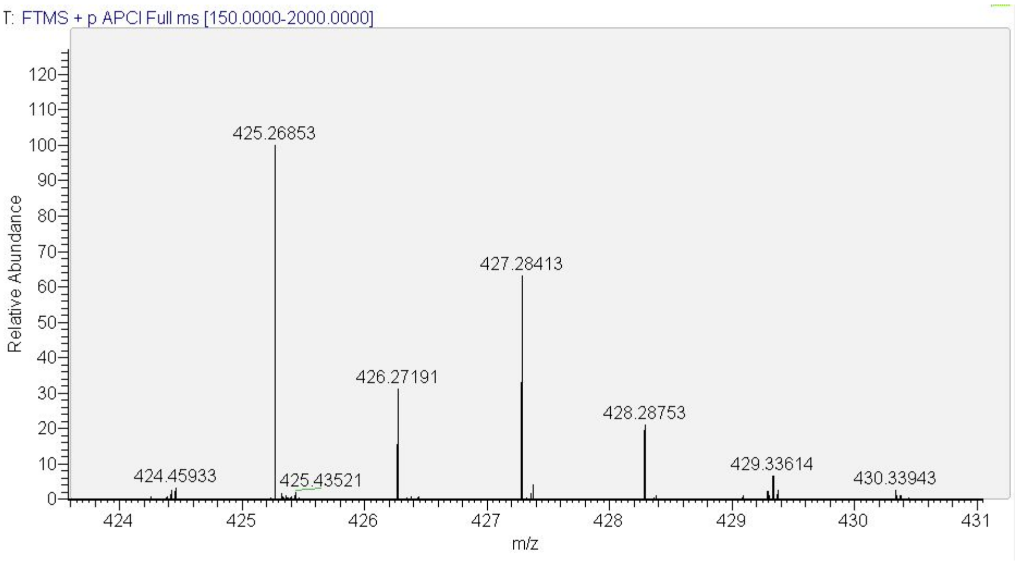

Figure S23. ^{1}H NMR spectrum of **5** in 800 MHz instrument, $CDCl_3$

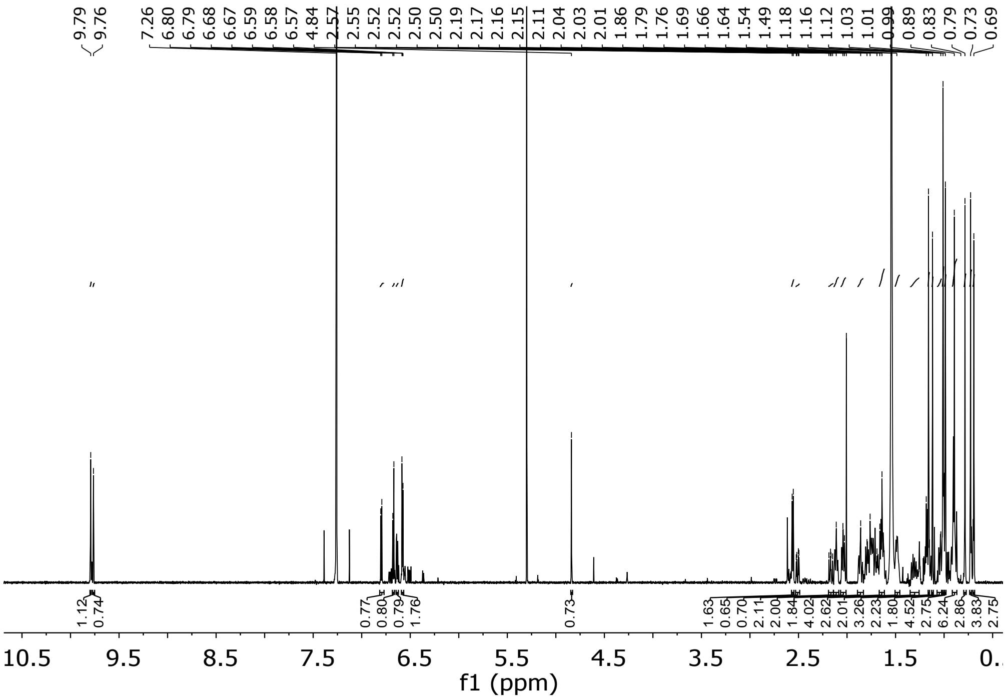

Figure S24. ^{13}C NMR spectrum of **5** in 800 MHz instrument, $CDCl_3$

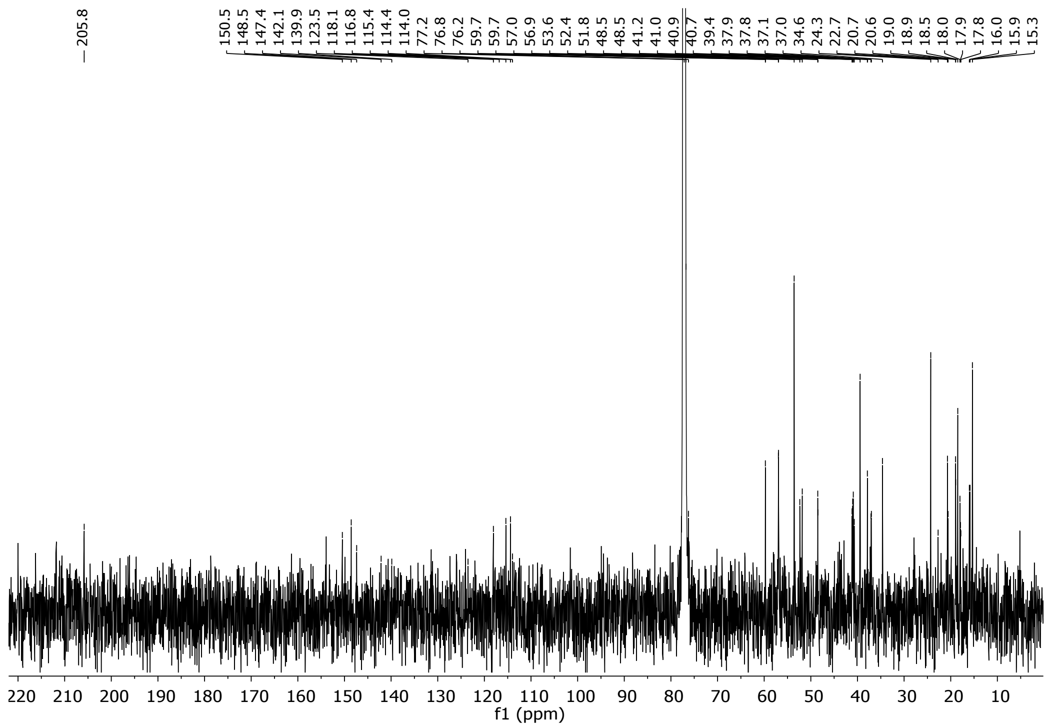

Figure S25. HSQC NMR spectrum of **5** in 800 MHz instrument, $CDCl_3$

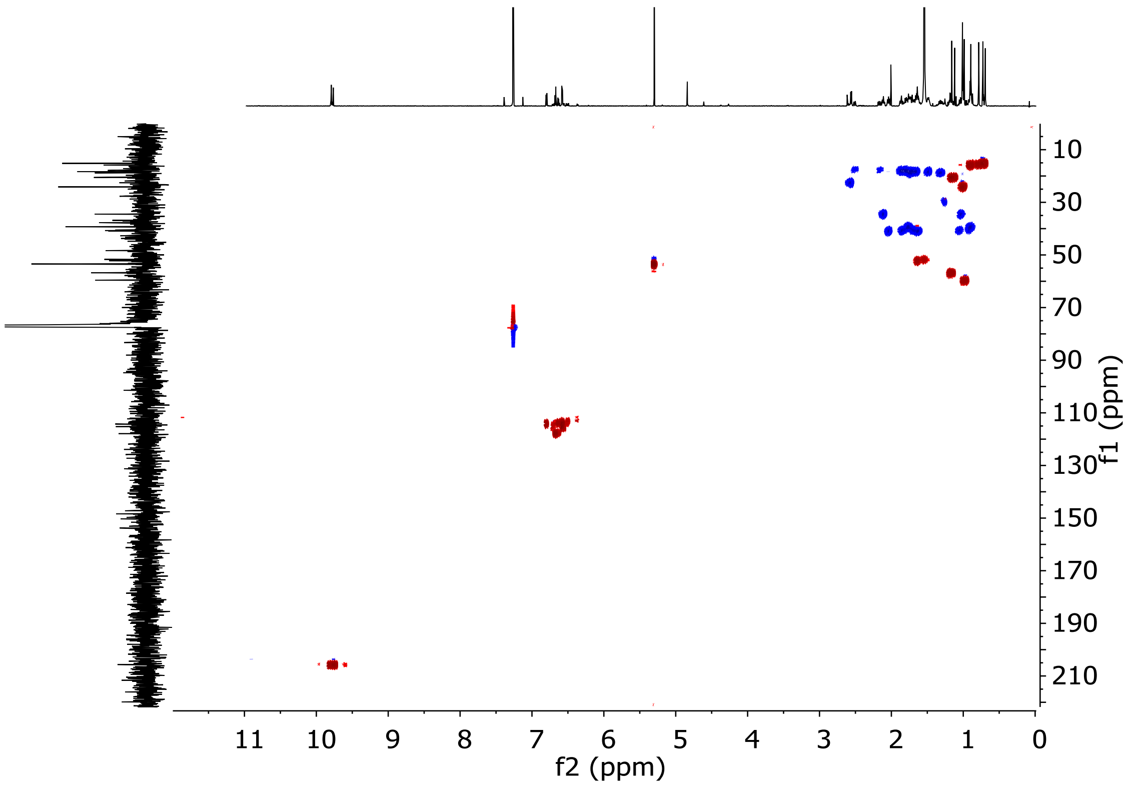

Figure S26. COSY NMR spectrum of **5** in 800 MHz instrument, $CDCl_3$

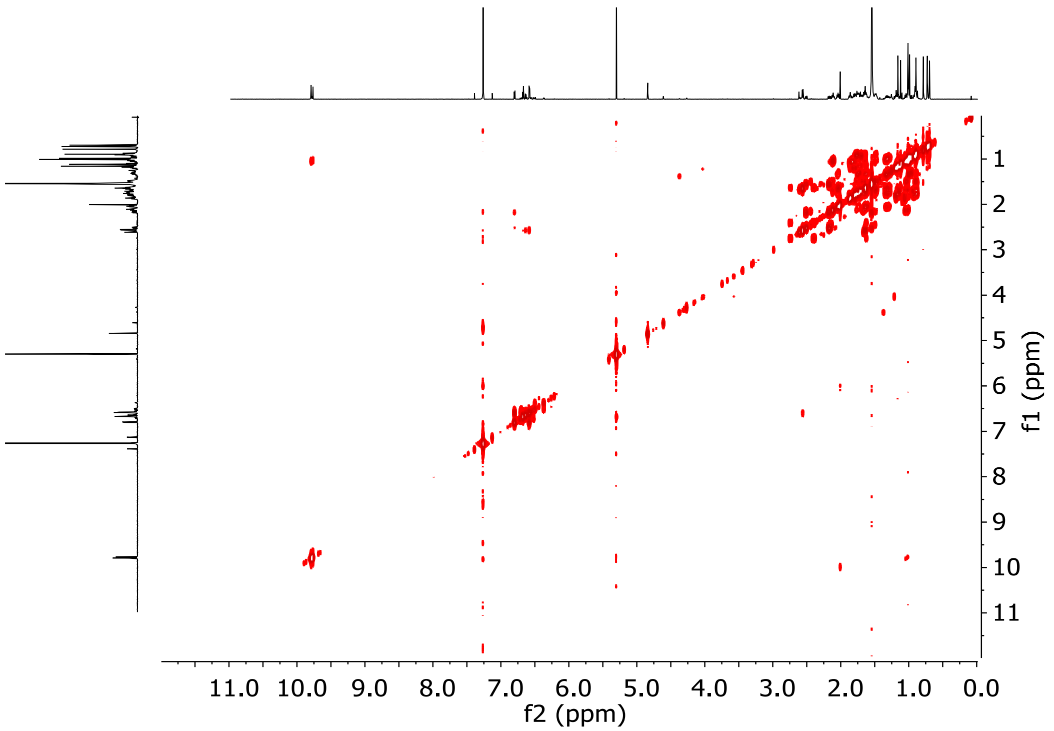

Figure S27. HMBC NMR spectrum of **5** in 800 MHz instrument, $CDCl_3$

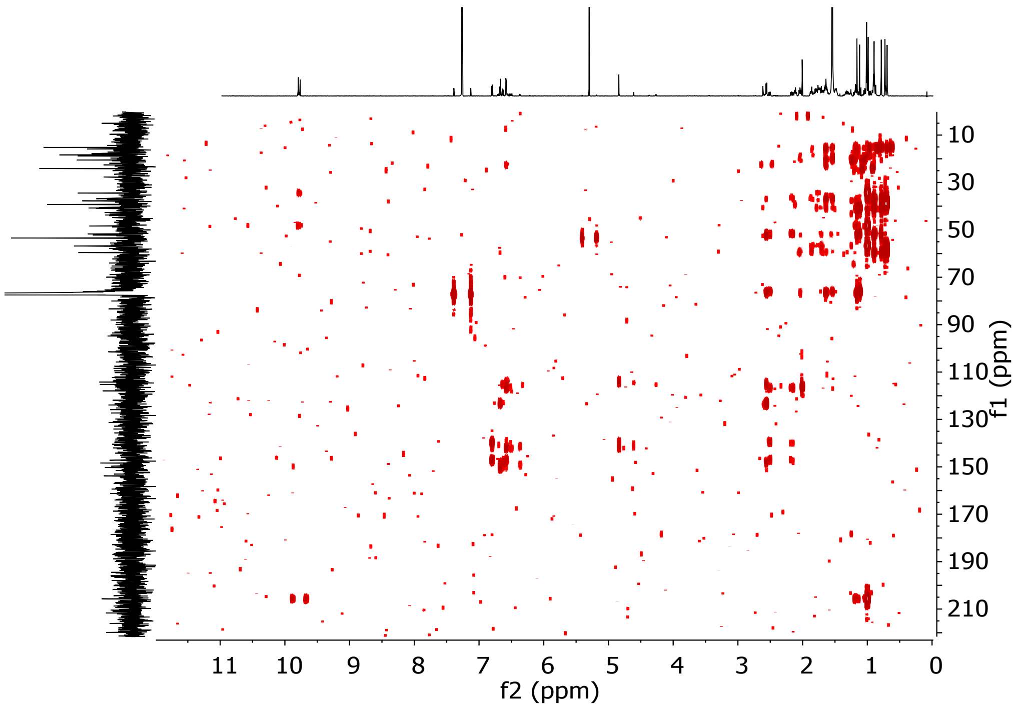

Figure S28. 1D ROESY NMR spectrum of **5** in 800 MHz instrument, $CDCl_3$

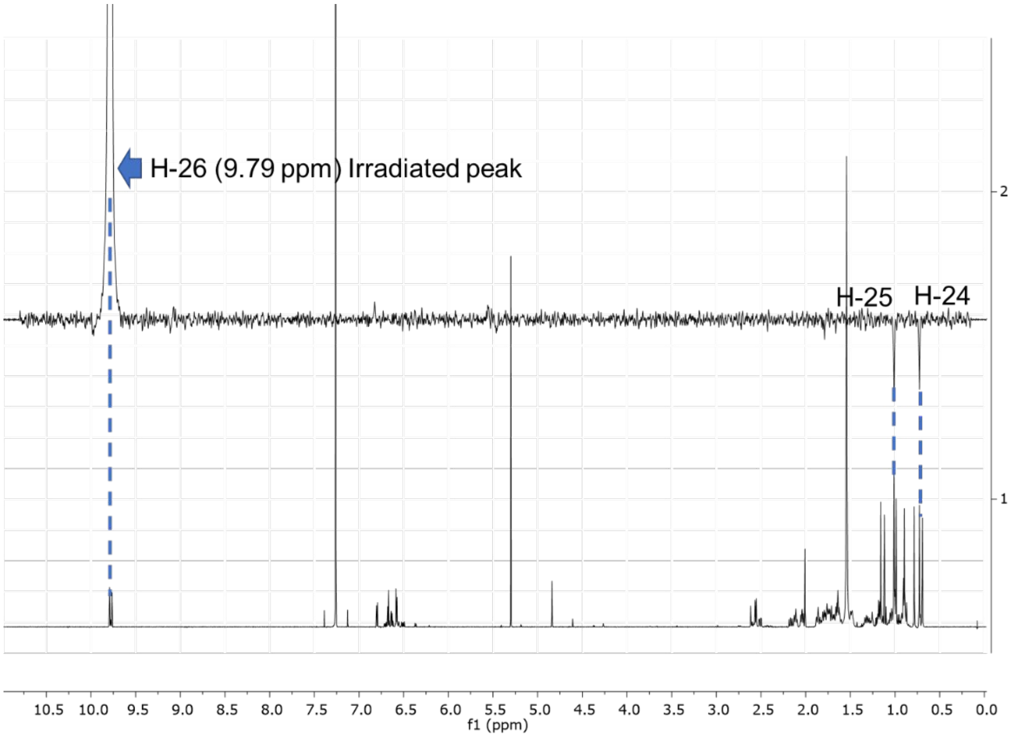

Figure S29. 1D ROESY NMR spectrum of **5** in 800 MHz instrument, $CDCl_3$

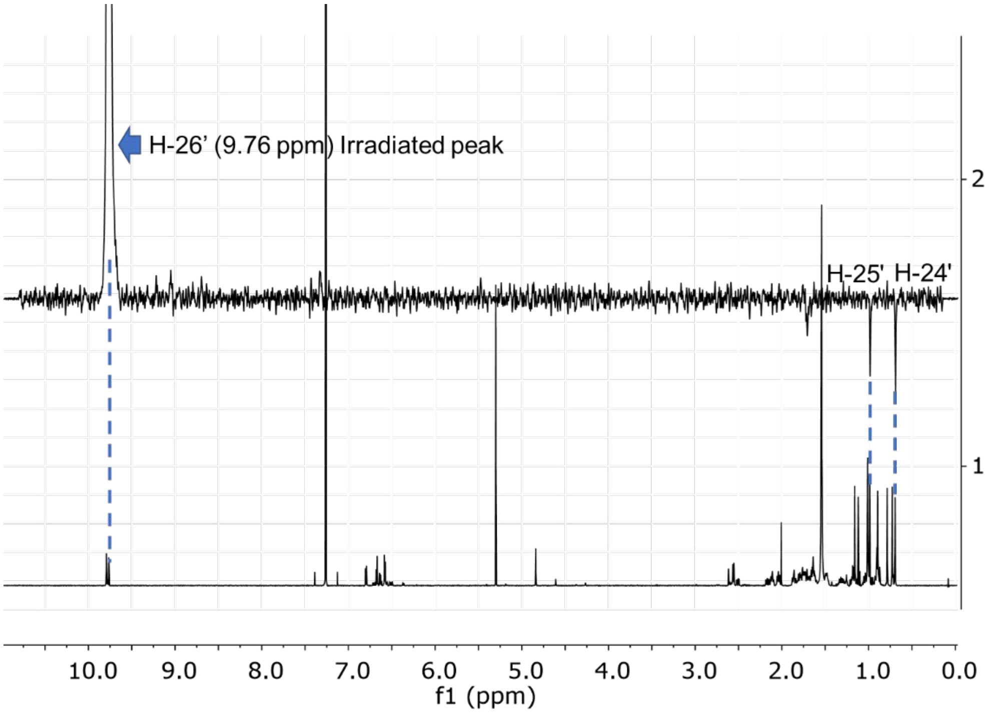

Figure S30. Positive ionization mode HRMS data for **5**

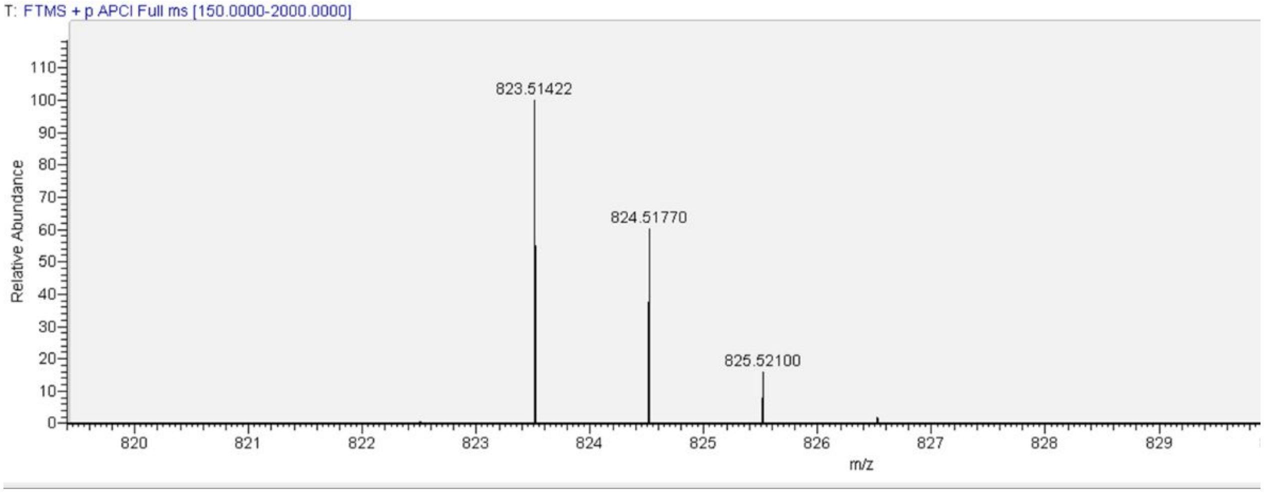

Figure S31. ^{1}H NMR spectrum of **6** in 800 MHz instrument, $CDCl_3$

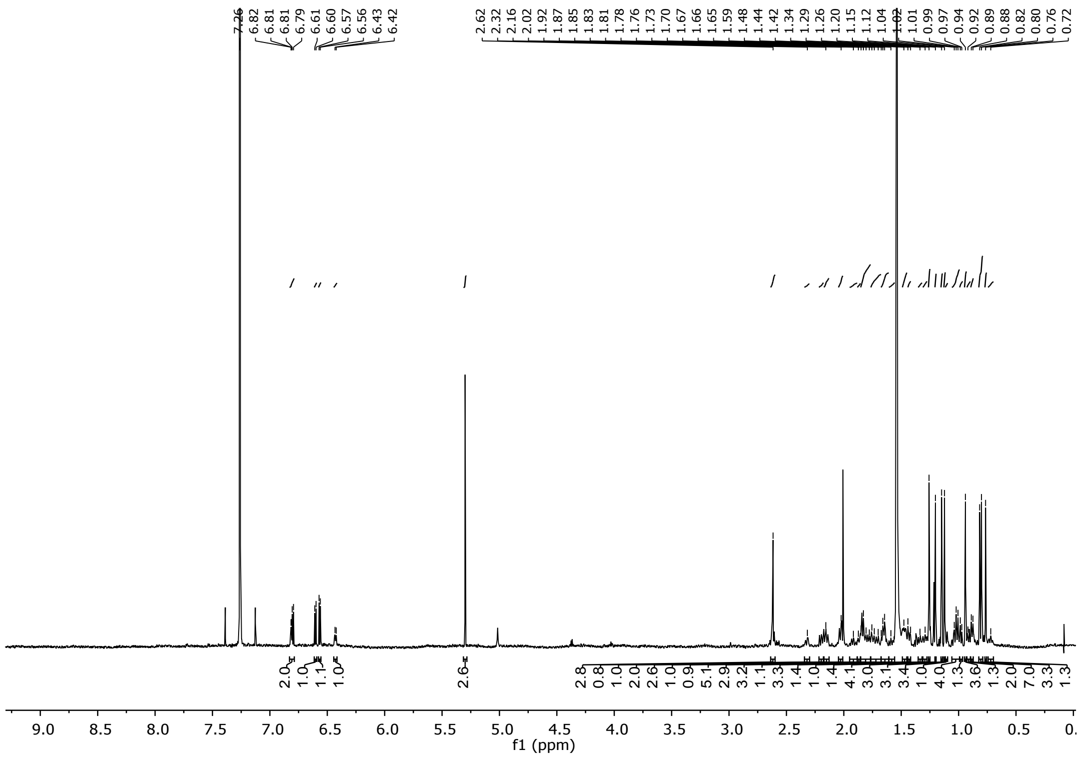

Figure S32. Positive ionization mode HRMS data for **6**

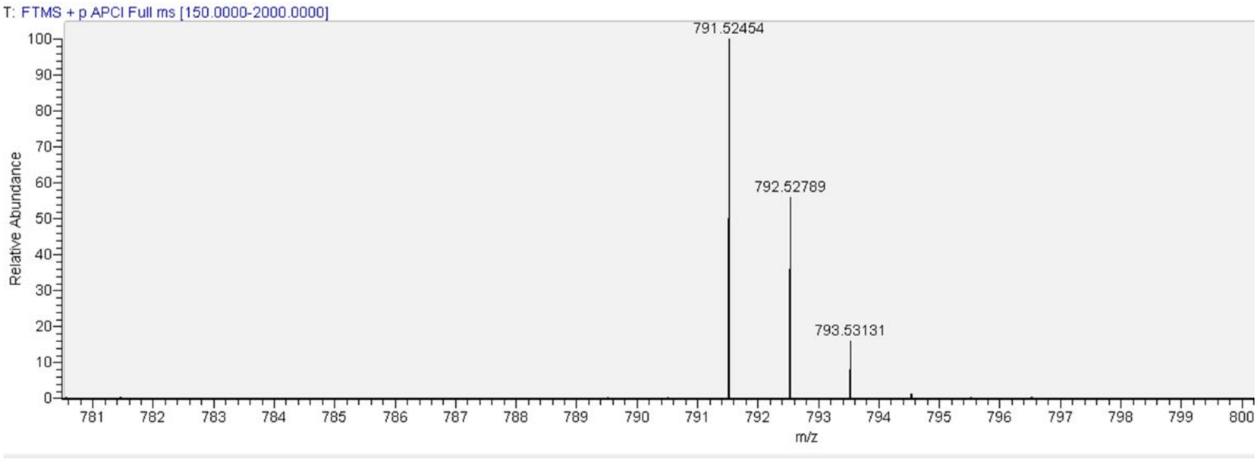

Figure S33. ^{1}H NMR spectrum of **7** in 800 MHz instrument, $CDCl_3$

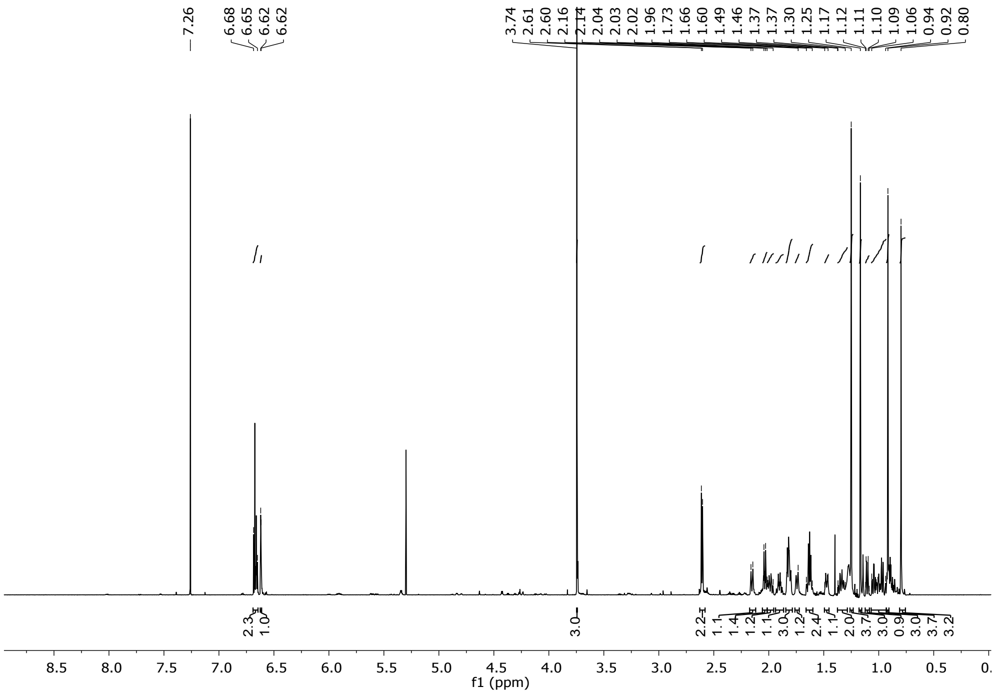

Figure S34. ^{13}C NMR spectrum of **7** in 800 MHz instrument, $CDCl_3$

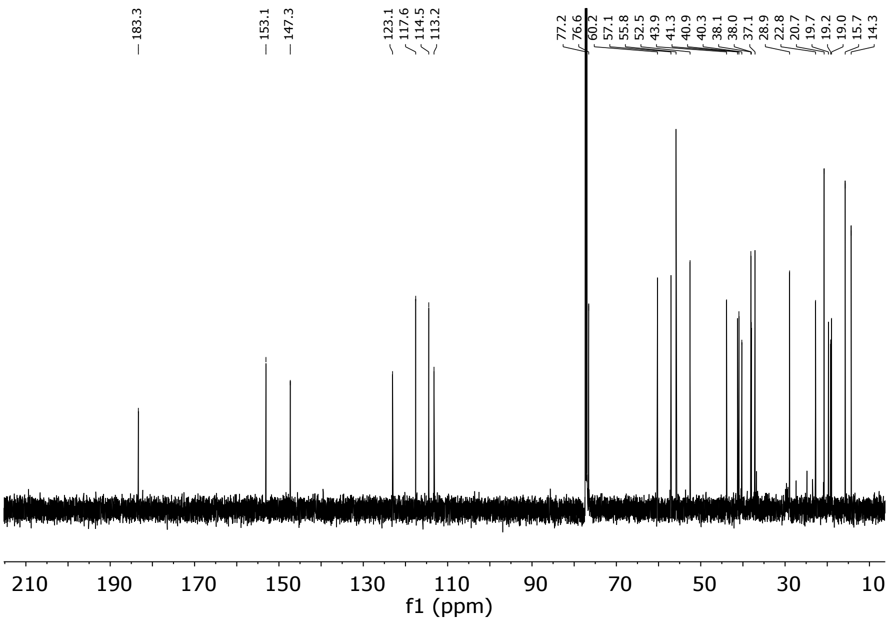

Figure S35. Positive ionization mode HRMS data for **7**

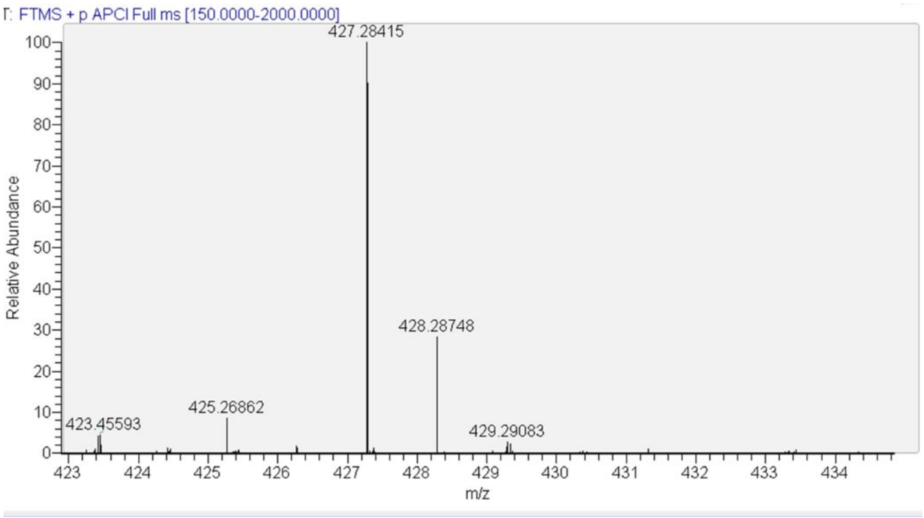

Figure S36. ^{1}H NMR spectrum of **8** in 800 MHz instrument, $CDCl_3$

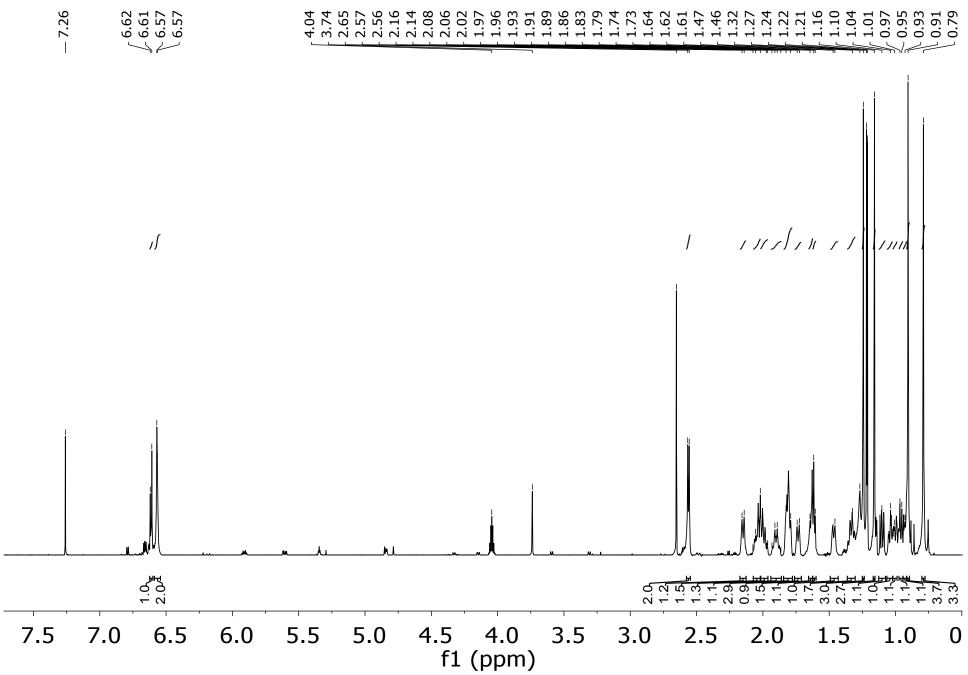

Figure S37. ^{13}C NMR spectrum of **8** in 800 MHz instrument, $CDCl_3$

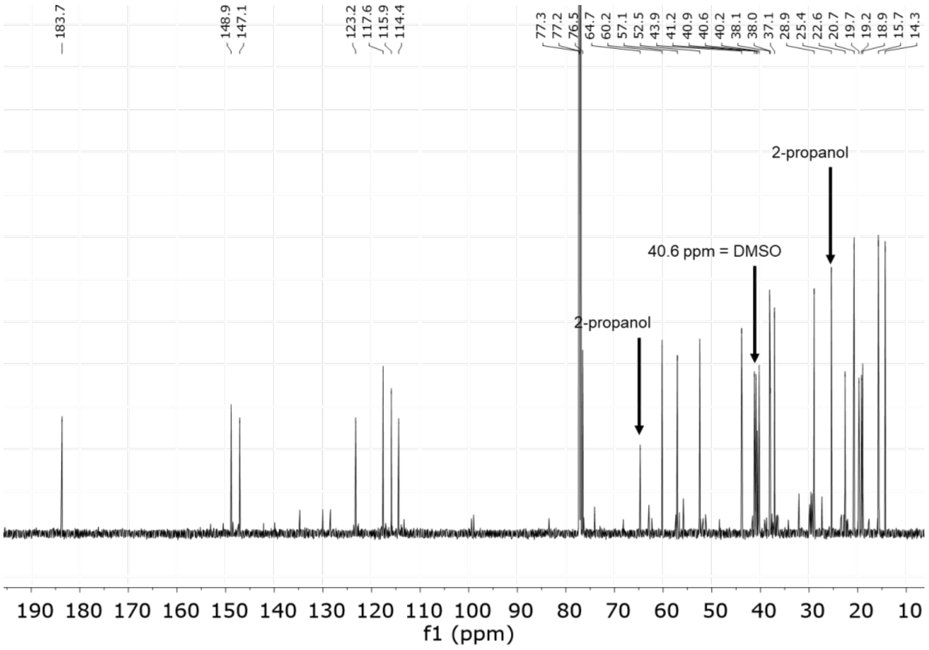

Figure S38. Positive ionization mode HRMS data for **8**

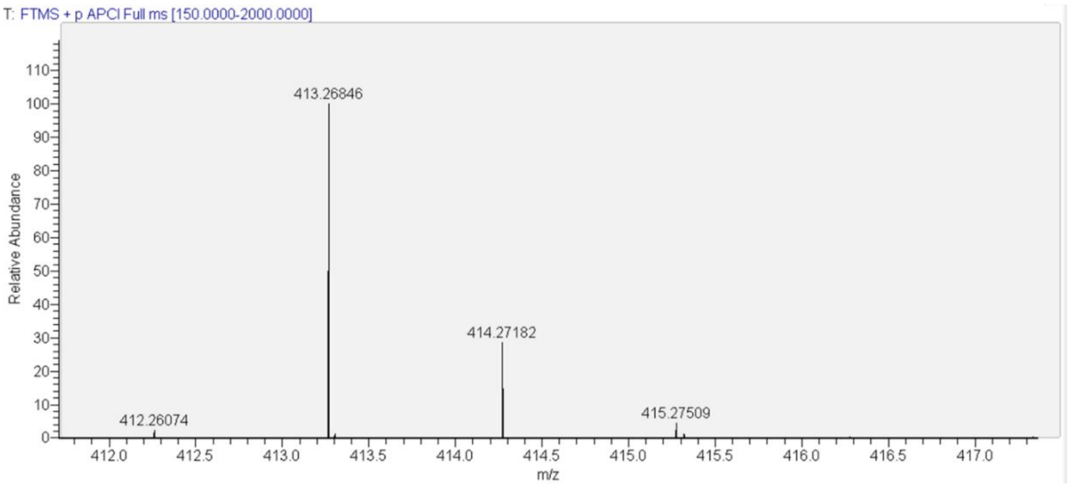

Figure S39. ^{1}H NMR spectrum of **9** in 800 MHz instrument, $CDCl_3$

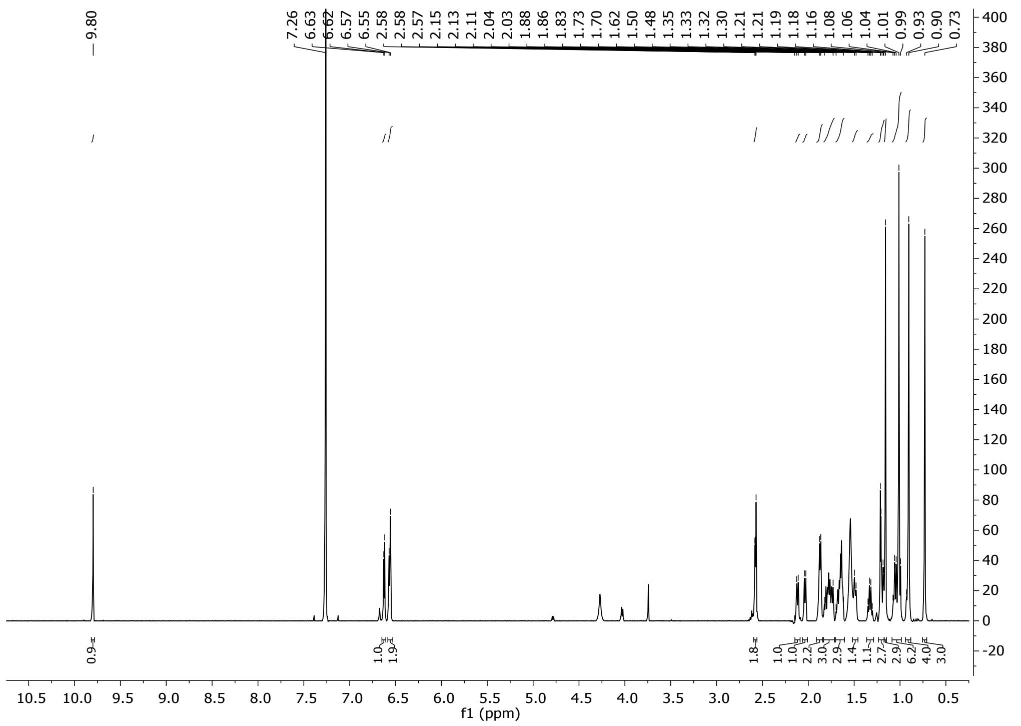

Figure S40. ^{13}C NMR spectrum of **9** in 800 MHz instrument, $CDCl_3$

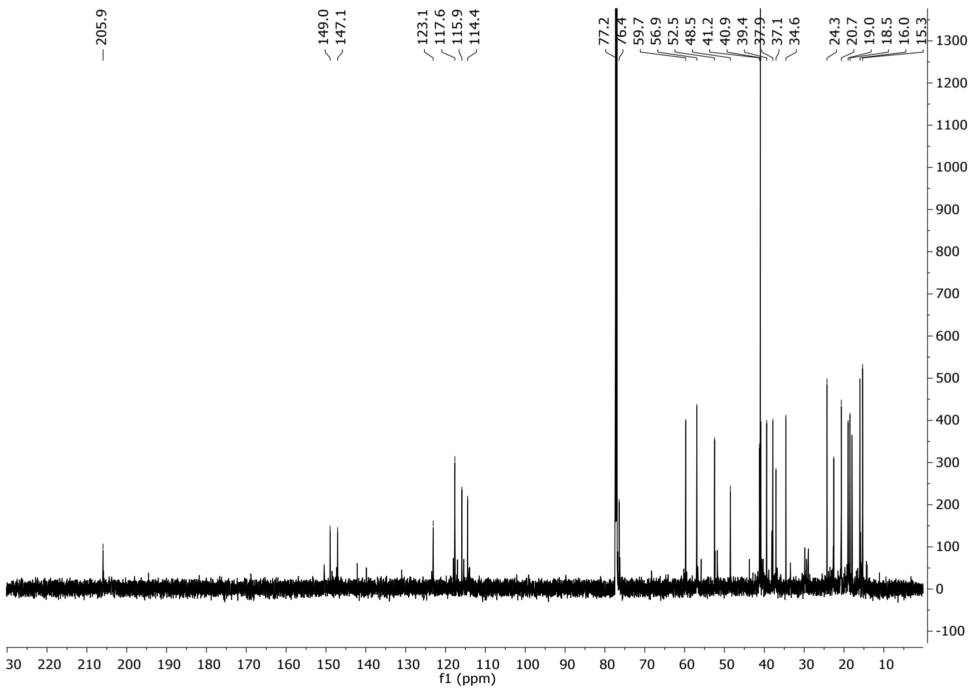

Figure S41. Negative ionization mode HRMS data for **9**

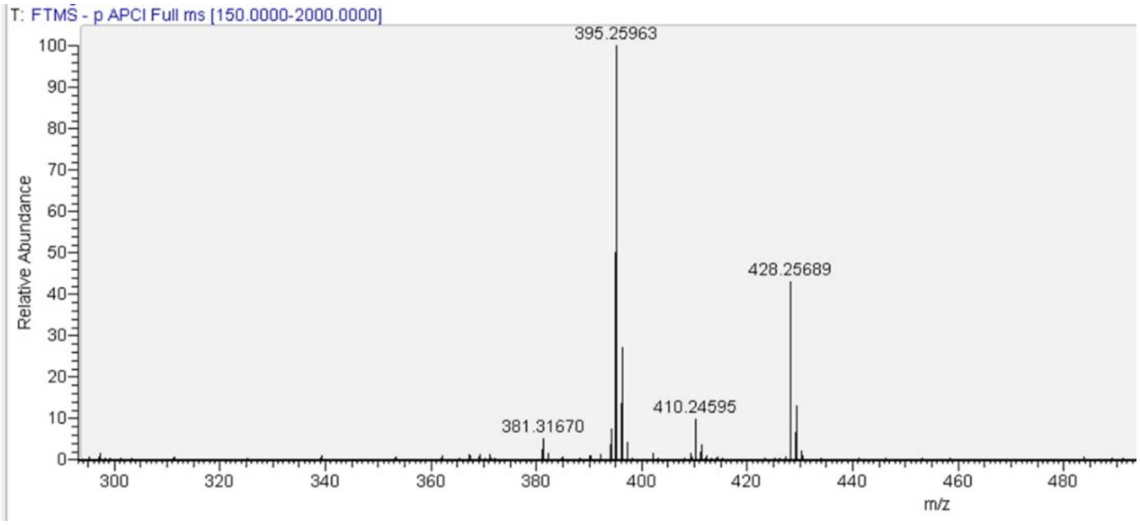

Figure S42. ^{1}H NMR spectrum of **10** in 800 MHz instrument, $CDCl_3$

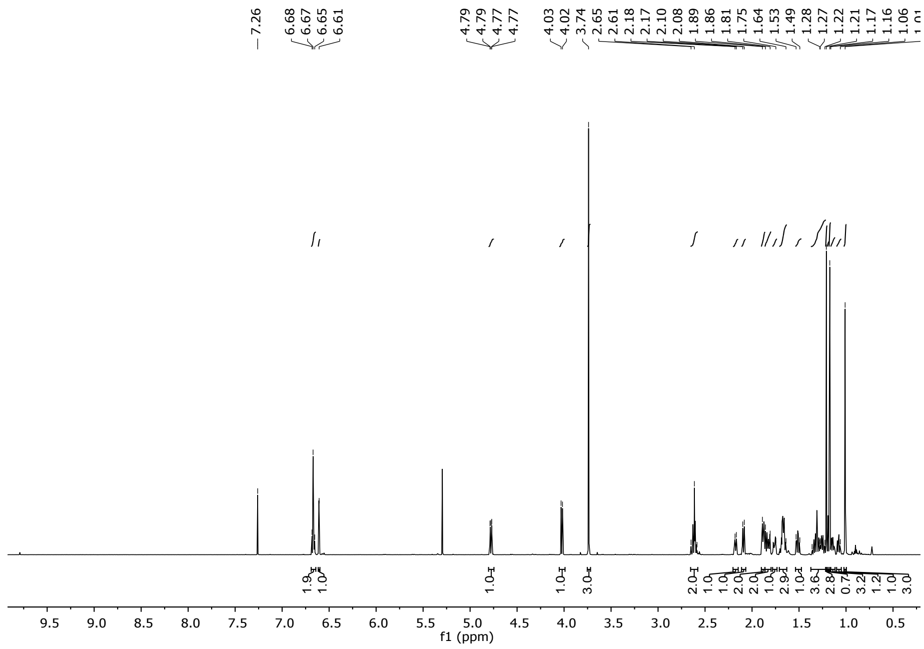

Figure S43. ^{13}C NMR spectrum of **10** in 800 MHz instrument, $CDCl_3$

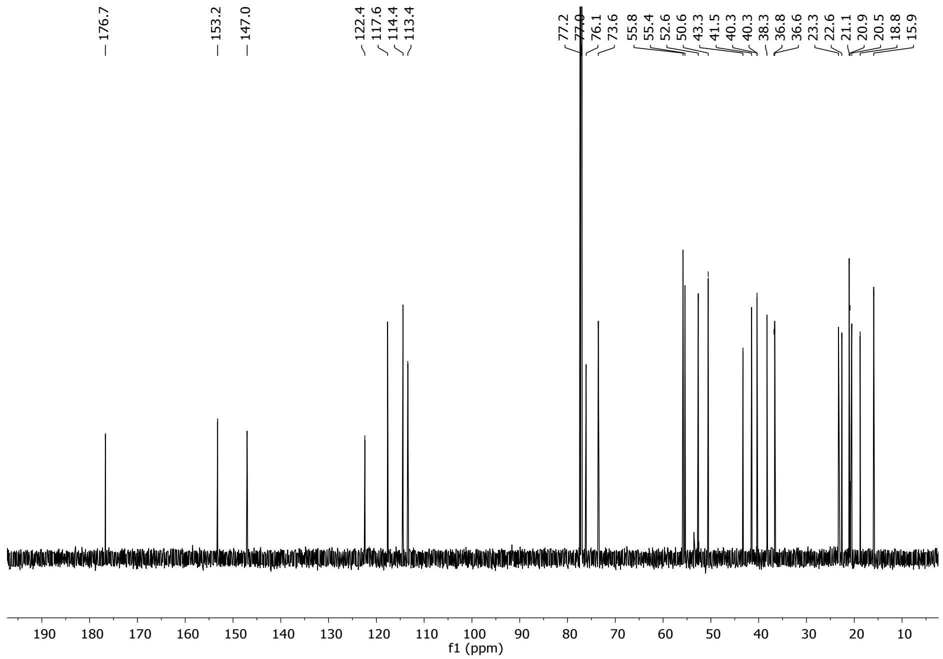

Figure S44. Positive ionization mode HRMS data for **10**

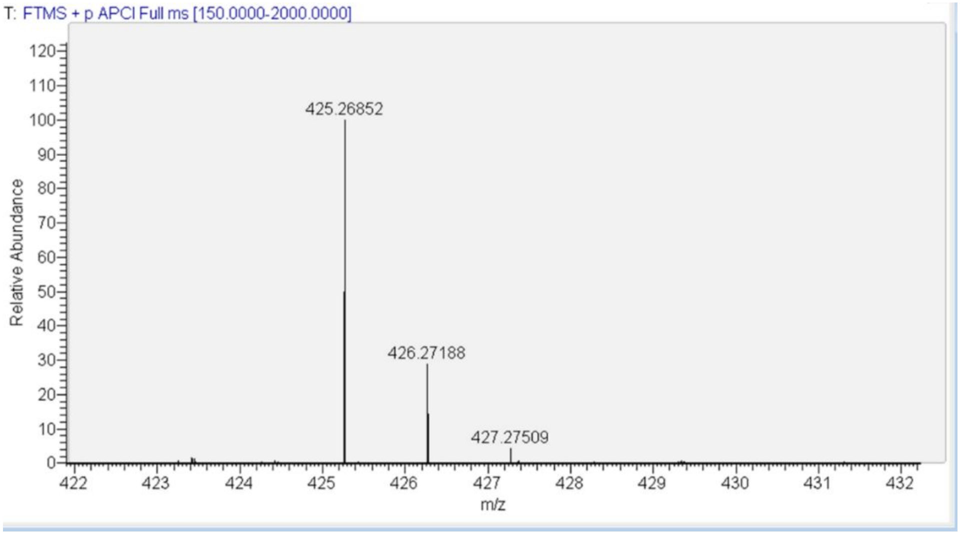

Figure S45. ^{1}H NMR spectrum of **11**–**12** in 800 MHz instrument, pyridine-d*5*

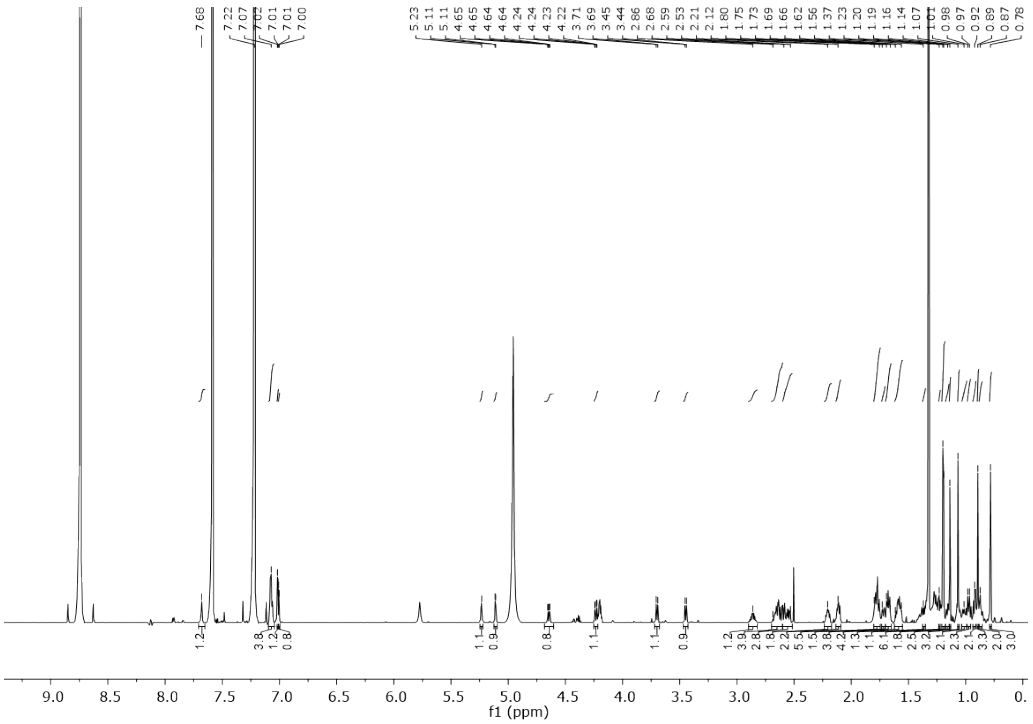

Figure S46. ^{13}C NMR spectrum of **11–12** in 800 MHz instrument, pyridine-d*5*

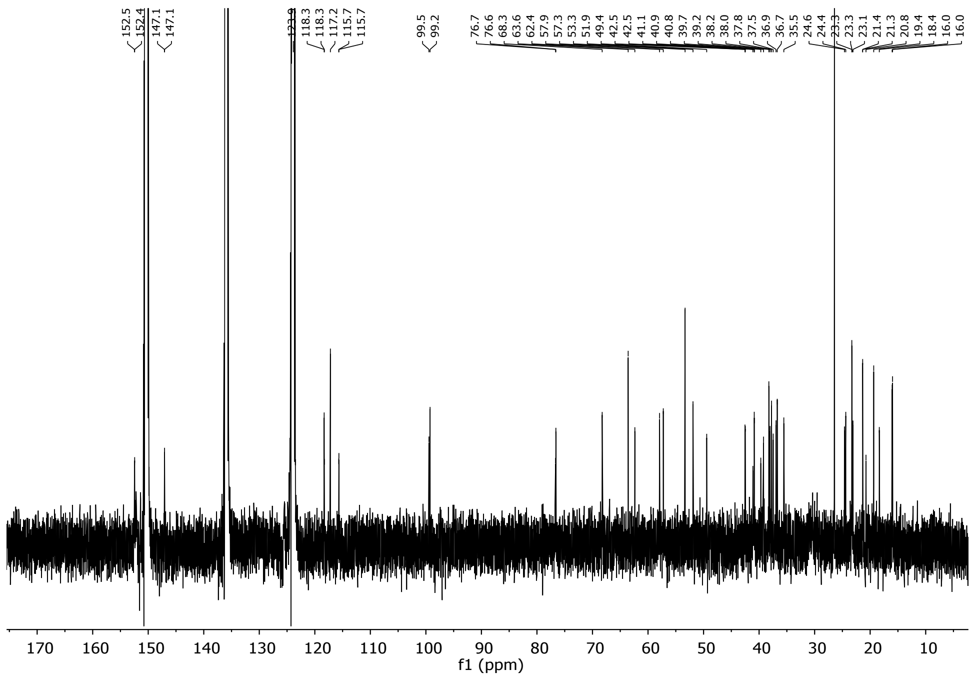

Figure S47. Positive ionization mode HRMS data for **11–12**

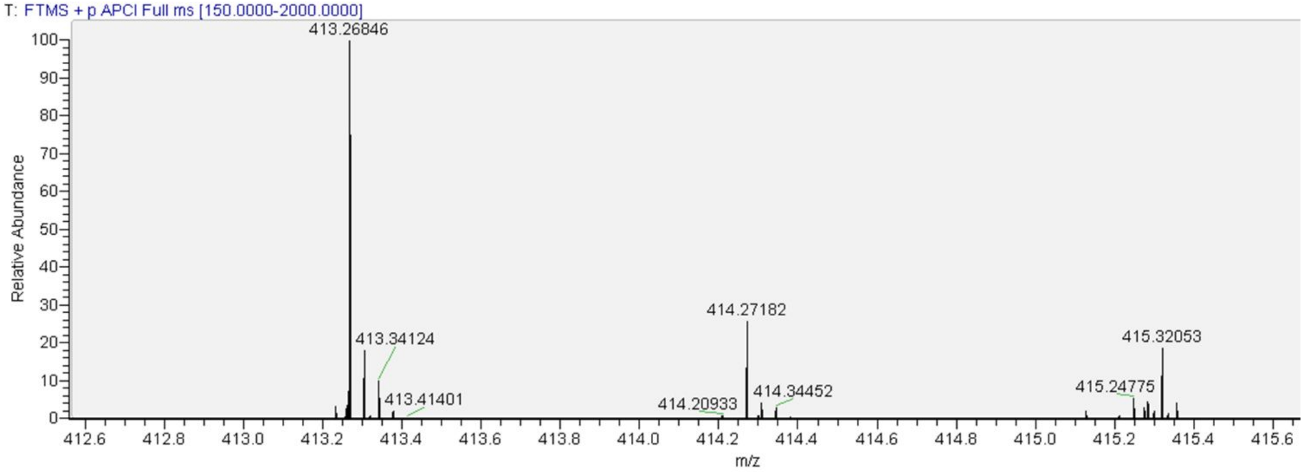

Figure S48. ^{1}H NMR spectrum of **13** in 800 MHz instrument, $CDCl_3$

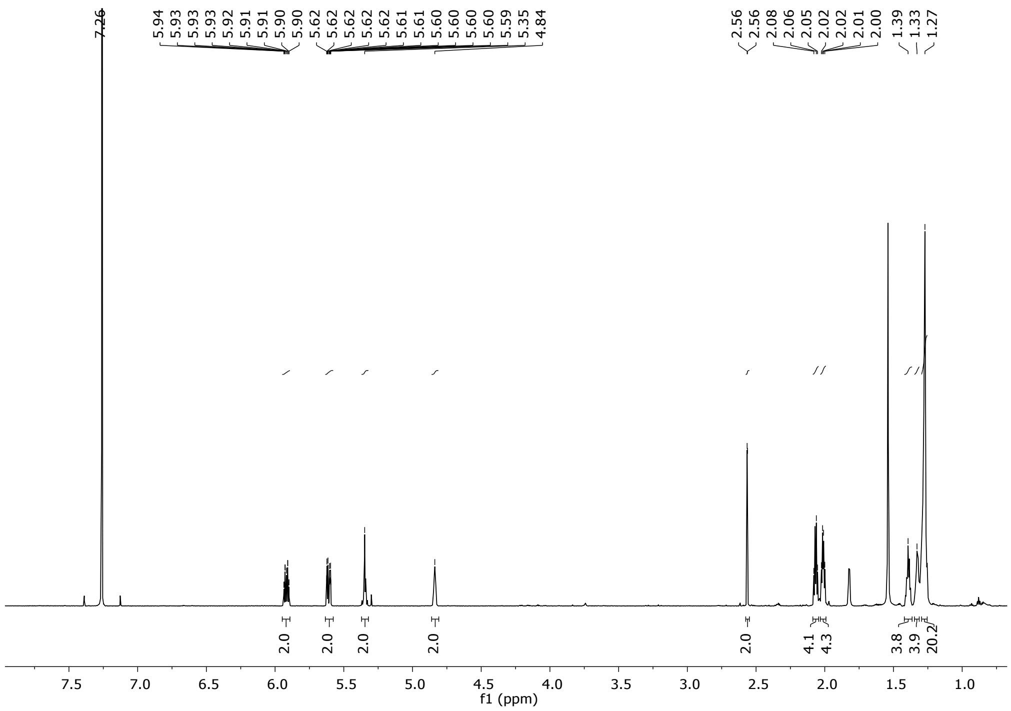

Figure S49. ^{13}C NMR spectrum of **13** in 800 MHz instrument, $CDCl_3$

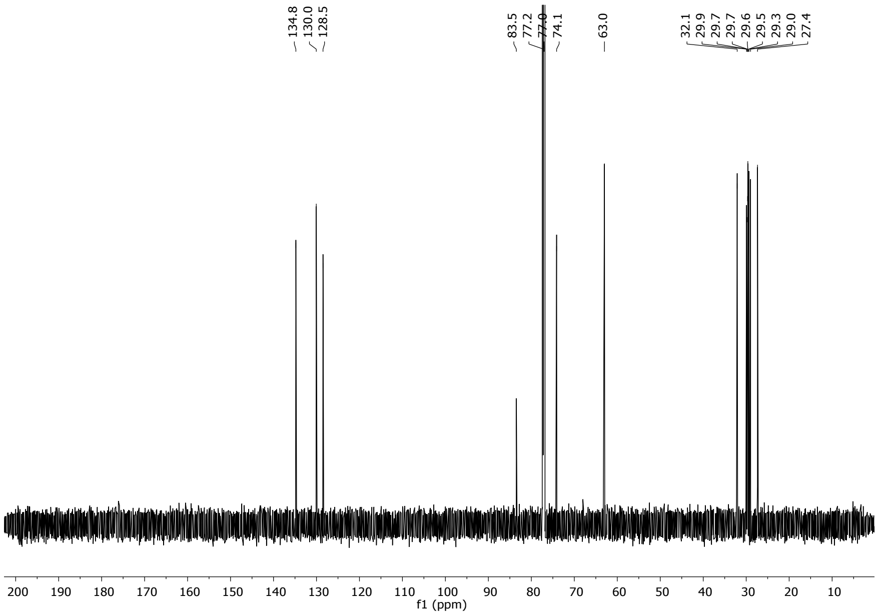

Figure S50. Positive ionization mode HRMS data for **13**

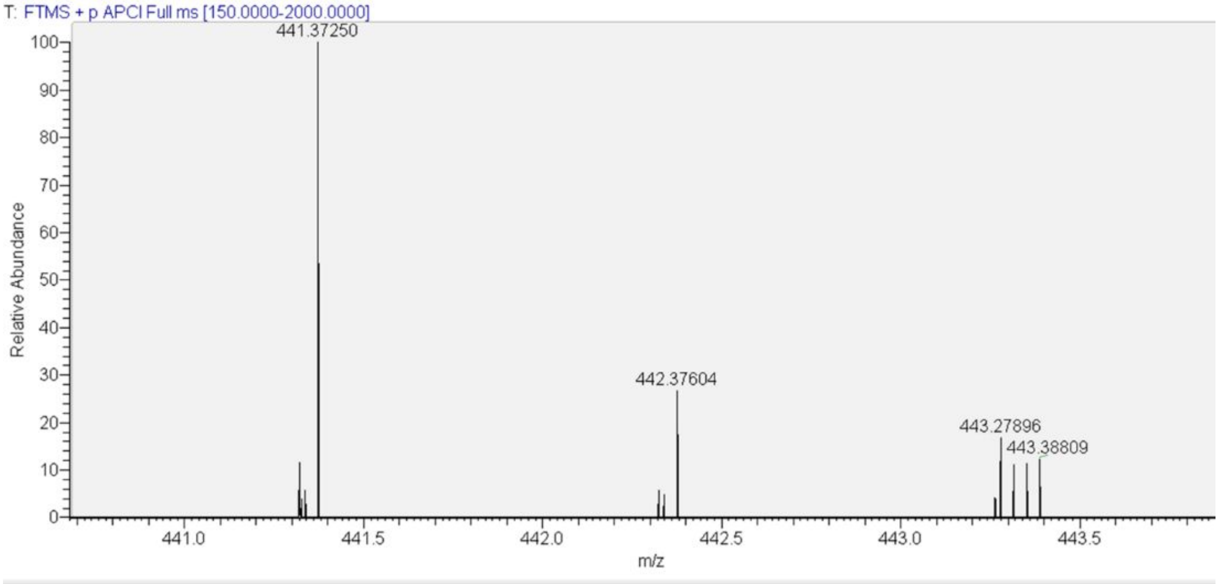

1. Balbin-Oliveros, M.; Edrada, R. A.; Proksch, P.; Wray, V.; Witte, L.; Van Soest, R. W. A new meroditerpenoid dimer from an undescribed Philippine marine sponge of the genus *Strongylophora*. *J. Nat. Prod.* **1998**, *61* (7), 948-952.
2. Yu, W.; Hjerrild, P.; Overgaard, J.; Poulsen, T. B. A Concise Route to the Strongylophorines. *Angew. Chem. Int. Ed.* **2016**, *55* (29), 8294-8438.
3. Liu, H.; Namikoshi, M.; Akano, K.; Kobayashi, H.; Nagai, H.; Yao, X. Seven new meroditerpenoids, from the marine sponge *Strongylophora strongylata*, that inhibited the maturation of starfish oocytes. *J. Asian Nat. Prod. Res.* **2005**, *7* (4), 661-670.
